# Recent Analytical Methodologies in Lipid Analysis

**DOI:** 10.3390/ijms25042249

**Published:** 2024-02-13

**Authors:** Ivana Gerhardtova, Timotej Jankech, Petra Majerova, Juraj Piestansky, Dominika Olesova, Andrej Kovac, Josef Jampilek

**Affiliations:** 1 Institute of Neuroimmunology, Slovak Academy of Sciences, Dubravska cesta 9, SK-845 10 Bratislava, Slovakia; ivka.gerhardtova@gmail.com (I.G.); timotej.jankech@gmail.com (T.J.); petra.majerova@savba.sk (P.M.); piestansky@fpharm.uniba.sk (J.P.); dominika.olesova@savba.sk (D.O.); andrej.kovac@savba.sk (A.K.); 2 Department of Analytical Chemistry, Faculty of Natural Sciences, Comenius University in Bratislava, Ilkovicova 6, SK-842 15 Bratislava, Slovakia; 3 Toxicological and Antidoping Center, Faculty of Pharmacy, Comenius University in Bratislava, Odbojarov 10, SK-832 32 Bratislava, Slovakia; 4 Department of Galenic Pharmacy, Faculty of Pharmacy, Comenius University in Bratislava, Odbojarov 10, SK-832 32 Bratislava, Slovakia; 5 Institute of Experimental Endocrinology, Biomedical Research Center, Slovak Academy of Sciences, Dubravska cesta 9, SK-845 05 Bratislava, Slovakia; 6 Department of Pharmacology and Toxicology, University of Veterinary Medicine and Pharmacy in Kosice, Komenskeho 68/73, SK-041 81 Kosice, Slovakia

**Keywords:** lipids, lipid analysis, sample treatment, liquid chromatography, mass spectrometry, data analysis

## Abstract

Lipids represent a large group of biomolecules that are responsible for various functions in organisms. Diseases such as diabetes, chronic inflammation, neurological disorders, or neurodegenerative and cardiovascular diseases can be caused by lipid imbalance. Due to the different stereochemical properties and composition of fatty acyl groups of molecules in most lipid classes, quantification of lipids and development of lipidomic analytical techniques are problematic. Identification of different lipid species from complex matrices is difficult, and therefore individual analytical steps, which include extraction, separation, and detection of lipids, must be chosen properly. This review critically documents recent strategies for lipid analysis from sample pretreatment to instrumental analysis and data interpretation published in the last five years (2019 to 2023). The advantages and disadvantages of various extraction methods are covered. The instrumental analysis step comprises methods for lipid identification and quantification. Mass spectrometry (MS) is the most used technique in lipid analysis, which can be performed by direct infusion MS approach or in combination with suitable separation techniques such as liquid chromatography or gas chromatography. Special attention is also given to the correct evaluation and interpretation of the data obtained from the lipid analyses. Only accurate, precise, robust and reliable analytical strategies are able to bring complex and useful lipidomic information, which may contribute to clarification of some diseases at the molecular level, and may be used as putative biomarkers and/or therapeutic targets.

## 1. Introduction

Lipids belong to a crucial group of biomolecules that participate in many vital cellular processes in various physio-pathological events; they are components of cell membranes, cell barriers, energy sources, and signal transduction, and serve as intermediates in signaling pathways [1,2]. Chemically, lipids are organic molecules with poor solubility in water [3]. Because of the high number of lipids, their classification is important. Dividing lipids into classes and subclasses is reliant on the lipid head group and the type of connection between aliphatic chains and the head group. The most common classification is according to their polarity. We distinguish non-polar, e.g., triacylglycerol (TAG) and cholesterol (Chol), polar lipids, e.g., ethanolamine glycerophospholipid (PE), choline glycerophospholipid (PC), inositol glycerophospholipid (PI) [4,5], and neutral, e.g., waxes and terpenes [6]. The best-known comprehensive classification system LIPID MAPS^®^ comprises more than 45,000 lipid structures in their database [4,5]. According to LIPID MAPS^®^, these biomolecules are classified into eight categories (Table 1), including fatty acyls (FA), glycerolipids (GL), glycerophospholipids (GP), sphingolipids (SP), sterol lipids (ST), prenol lipids (PR), saccharolipids (SL), and polyketides (PK) [7]. Most lipid classes contain a number of molecules that differ in terms of their stereochemical properties and composition of fatty acyl groups. These differences in lipid species and their homeostasis are involved in various pathological conditions [8]. Disruption of lipid homeostasis can lead to problems in living organisms, such as cardiovascular diseases, diabetes, chronic inflammation, neurological disorders or neurodegenerative diseases such as Alzheimer’s disease (AD) [1,9].

The profile of lipid classes found in a cell, organelle or tissue refers to the lipidome, while lipidomics represents lipid profiling in biological systems [11]. Lipidomics, as a part of lipid analysis, is a quickly growing tool in the exploration of lipid metabolism, the search for new biomarkers and the discovery of medicinal targets of lipid-related diseases [12,13].

Due to the complex structures of lipids and the large number of lipid species, analysis is more demanding, and thus all analytical steps, including sample preparation, separation, detection, data processing and interpretation, must be considered and verified for reliable identification and/or quantification of different lipid species from complex matrices [14,15]. The development of analytical methodologies is an emerging field, seeking to fulfill the high requirements of analysis results. For identification and quantification of lipids in various matrices, the most dominant are MS-based methods, which can be used without prior separation of lipids (direct MS) or in conjunction with appropriate separation technique, mostly liquid chromatography (LC) [13]. These methods enable accurate identification of lipid changes at the level of individual classes, subclasses and types of molecules [11,13]. Two approaches in lipid analysis are currently used: targeted and untargeted (or non-targeted). Both approaches have their own advantages and disadvantages [8]. Absolute concentrations of known metabolites (1–100 metabolites, depending on the number of investigated analytes), thanks to the use of standards and calibration curves of selected metabolites, provide a targeted approach [16,17]. An untargeted approach can cover the detection of lipids in the hundreds to low thousands using a combination of separation and detection modes. Semi-quantitative data are obtained by untargeted analysis, where each lipid peak area is reported (instead using absolute concentration of each analyte) [16,18].

In rapidly evolving fields such as lipid analysis, staying current is important. Advances in sample preparation and instrumentation, characterized i.e., by increased MS resolution and enhanced sensitivity in new MS devices, present opportunities for analyses that are not only more sensitive and accurate but also faster, enabling the monitoring of numerous analytes in a single run. Consequently, publication of review articles regularly becomes essential to provide authors in this field with a contemporary understanding of the state-of-the-art. To our best knowledge, a comparably comprehensive overview of lipid analysis has not been published in the past five years.

This article is focused on overview of latest (last five years) advances in lipid analysis. In this review, the advantages and limitations of extraction techniques are discussed, and analytical techniques used in lipid analysis are critically reviewed. Furthermore, analysis of obtained data is included.

## 2. Sample Pretreatment

Sample pretreatment comprises all actions performed with the sample from its delivery to the laboratory to its analysis. That is why it is the most important and the most critical step of the entire analytical procedure in chemical analysis, especially in lipid analysis. Since lipids are susceptible to oxidation or their hydrolysis may occur (depending on the matrix), it is necessary to process the sample as soon as possible or freeze it at −80 °C or lower [1,2,12,19].

Sample treatment used in lipid analyses strategies is typically accompanied with extraction procedures. Lipid extraction is often preceded by preparation of the analyzed sample. The sample preparation step can include mechanical, biological, chemical or physical operations, which are included before the extraction step. When using these techniques, a better penetration of the solvent into the matrix is achieved. As such, the robustness of the entire method could be increased [11,20]. The sample preparation method is chosen mainly based on the physical state of the sample. While the treatment of liquid samples (e.g., plasma or urine) is relatively simple, the treatment of solid samples, such as tissues, is more difficult and, in most cases, requires reduction or disruption of solid sample particles and homogenization [21]. Other sample preparation methods include physical and mechanical operations such as bead milling, hydrodynamic cavitation, ultrasonication, autoclaving and microwave irradiation, biological procedures, i.e., use of enzymes, or chemical procedures, e.g., osmotic shock of cells [11,22,23,24]. Another aim of sample preparation in lipid analysis is the improvement of lipid stability by adding additives or antioxidants to the sample or treating the sample by flash freezing or heat [12]. Whether subsequent lipid extraction will be effective depends on selecting an appropriate sample preparation method [11]. 

### 2.1. Extraction of Lipids

Several procedures are currently available for the extraction of lipids from different matrices. Whether the result of the analysis will be quantitative/qualitative depends, among other things, on the appropriately chosen extraction method. Differences in lipid structure, molar weight and polarity make this part of sample pretreatment very challenging [7,11,25]. The choice of extraction method is also dependent on the type of analyzed sample or the properties of lipids: (*i*) sample origin (human, animal, plant, food), (*ii*) physical state (fluid, tissue), (*iii*) physicochemical properties of lipids (polarity). Polarity of lipids is the key factor in the selection of extraction solvents. Another crucial factor affecting the extraction procedure is the complexity of matrices. Therefore, there is a need to minimize the matrix effect. This step includes selective removal of other interfering non-lipid components from the sample [1,26]. Typical interferents, which need to be removed from biological samples such as serum or tissue, are represented by proteins. For this purpose, it is necessary to implement a simple operation known as protein precipitation (PP) and choose a solvent that is also suitable for the extraction of lipids [27]. However, in most cases, PP and the lipid extraction itself are two separate steps. The simplest extraction method is single organic solvent extraction (SOSE) using polar solvents like acetonitrile (ACN) or methanol (MeOH), which is limited in the extraction of neutral or non-polar lipids [21]. However, one-phase extraction (OPE) is an analog of SOSE but includes the use of two or more miscible solvents creating one phase, e.g., butanol (BuOH):methanol (MeOH) in ratio 3:1, known as the BUME method [28]. Similarly, the same organic solvent mixture (BuOH:MeOH) at the 1:1 solvent ratio can also be used as an efficient lipid extraction environment [29]. OPE becomes very effective for the extraction of less polar lipids and nowadays is gaining more and more popularity, especially thanks to its simplicity [30,31].

**Figure 1:**
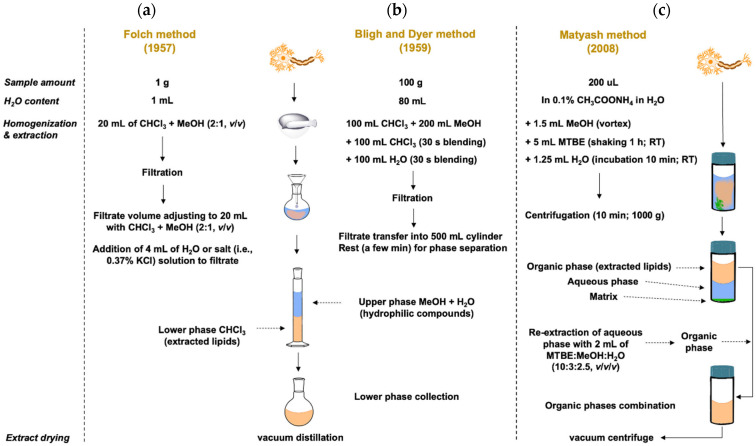
Illustrated comparison of conventional original forms of (**a**) Folch method; (**b**) Bligh and Dyer method; (**c**) Matyash method. Modified by [11]. Copyright 2021 MDPI.

Another preferred method used in lipid sample pretreatment is liquid-liquid extraction (LLE) [1]. All currently used protocols (regardless of the nature and origin of the sample) are based on the Folch [32] and Bligh and Dyer methods [11,33,34], which were originally developed for lipids extraction from tissues. These two methods are considered as the “gold standard” in lipid extraction. Recently, many modifications of these methods are known, either in an attempt to increase the extraction efficiency or in an attempt to replace the toxic solvents used in these methods: chloroform (CHCl_3_): MeOH (in a ratio of 2:1 in Folch method and 1:2 in Bligh and Dyer method, respectively) [34,35]; less toxic solvents include propanol, isopropanol (IPA), ethyl acetate (EtOAc), ethanol (EtOH) or their combination [36,37]. Another modification is the use of methyl *tert*-butyl ether (MTBE), i.e., Matyash’s method [38]. In this method, lipids are extracted in the upper (organic) phase; this phase is easily collected and represents an advantage over the Bligh and Dyer or Folch methods, where lipids are in the lower (CHCl_3_) phase. The disadvantage of MTBE is the volatility of MTBE; therefore, it is necessary to ensure the reproducibility control of the extraction [11,39]. A simple comparison of the Bligh and Dyer, Folch and Matyash methods is illustrated in Figure 1.

**Figure 2:**
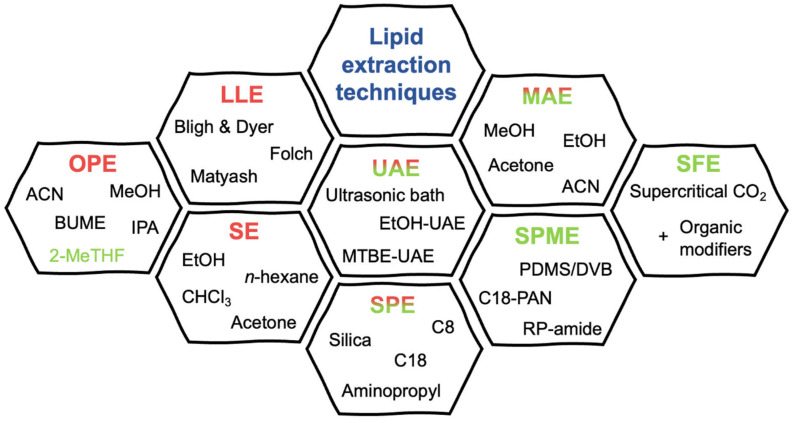
Overview of extraction techniques (with representative examples of solvents, phases, etc.) used in lipid analysis; individual techniques are colored according to their “greenness”: Red (OPE, LLE, SE)—conventional extraction techniques with possibility of using greener solvents, such as 2-MeTHF; Red-to-green gradient (SPE; UAE; MAE)—greener techniques requiring usage of organic solvents; Green (SPME; SFE)—green extraction techniques. C18-PAN: C18-polyacrylonitrile; EtOH-UAE: ethanol-ultrasound-assisted extraction; MAE: microwave-assisted extraction; MTBE-UAE: methyl-*tert*-butyl ether-ultrasound-assisted extraction; PDMS/DVB: polydimethylsiloxane/divinylbenzene; RP-amide: reversed-phase-amide; SE: Soxhlet extraction; SFE: supercritical fluid extraction; SPE: solid phase extraction; SPME: solid phase microextraction.

In 2019, Wong et al. provided a comparison of the modified BUME method introduced by Alshehry et al. [29] with classical Folch and Matyash’s methods. BUME method reached comparable results in terms of reproducibility and recovery and in its ability to detect a wide range of lipid classes without using chloroform [40]. Another chloroform-free method was published recently, in which the three-phase liquid extraction was introduced for lipidomic workflows. A mixture of distinct liquid phases consists of hexane, methyl acetate, ACN, and water. Using this technique, polar and neutral lipid fractions are made. Separating lipids into two distinct organic phases leads to less complex extracts; this comes with other advantages, such as lower background or decreased ion suppression in comparison with the use of the modified Bligh and Dyer method. On the other hand, the analysis time is doubled if both lipid profiles are needed [41]. In addition to the listed less toxic solvents, there is an effort to incorporate green solvents, e.g., terpenes [42], cyclopentyl methyl ether (CPME) [43], or a combination of 2-methyl tetrahydrofuran (2-MeTHF) and CPME. Unfortunately, this solvent system showed significantly lower yields of lipids extraction than classic Bligh and Dyer and Folch protocols [44]. On the other hand, another combination of green solvent system consisting of 2-MeTHF:isoamyl alcohol:H_2_O showed higher yields of lipids compared to classic extraction methods [11]. However, the developed extraction method was significantly more expensive than the classic extraction method, which is highly undesirable and makes the position of green extraction solvents more difficult in competition with cheaper non-green toxic solvents such as chloroform or MeOH [11,42]. An overview of extraction techniques (with representative examples of solvents, phases etc.) used in lipid analysis is shown in Figure 2.

**Figure 3:**
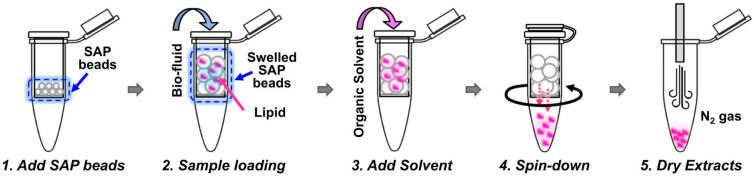
Illustration of novel modified method using spin column filled with SAP beads. Adapted from [46]. Copyright 2023 SpringerOpen.

Other extraction techniques include solid phase extraction (SPE), which can be used for lipid extraction, especially in targeted lipidomics, where selected groups of lipids can be selectively extracted [27]. However, it is more often used as a clean-up technique after the previous LLE extraction. Suitable and commonly used SPE columns for more polar lipids are silica and aminopropyl. For non-polar lipids, reversed-phase columns (C8 or C18) are commonly utilized. A miniaturized parallel of SPE is solid phase microextraction (SPME), which is usually used prior to GC analysis [21]. Based on SPE principles, the simplified method for lipid extraction using superabsorbent polymer powders (SAP) was introduced a few years ago [45]. This technique was recently modified by [46]. The novel modified method utilized a spin column filled with SAP beads (Figure 3). The method was reproducible, sensitive and timesaving, and it showed an especially high extraction efficiency of lipids. A significantly lower (seven times) limit of detection (LOD) of PC 17:0/17:0 spiked into plasma in comparison with conventional methods was achieved. Moreover, a considerably lower 5-day period relative standard deviation (RSD) values for variability (inter- and intra-day) in comparison with previous SAP and Matyash methods were reached. According to these results, the modified SAP method could be a promising approach to lipid analysis [46].

Ultrasound-assisted extraction (UAE) [47], like SPE, is more often used in combination with OPE or LLE to increase the extraction efficiency [21]. An example of such an extraction was performed a few years ago. Xie et al. [48] developed the OPE-UAE method for egg yolk lipids profiling. IPA was used as an extraction solvent. The optimized conditions of the extraction process were as follows: liquid-solid ratio was 5.2:10 (*v*/*w*), ultrasound power was 182 W and time was 43 min. This approach enabled the characterization of 646 lipid species. Furthermore, compared with conventional methods (Folch and Matyash), the IPA-UAE method showed improved extraction efficiency and particularly solved the main drawback of conventional methods, i.e., some lipids poor recovery [48].

Other less-used extraction techniques are microwave-assisted extraction (MAE), which can cause the decomposition of thermolabile analytes [21], or Soxhlet extraction (SE), which is commonly performed using hexane [49]. SE is controversial in the world of lipid analysis. On the one hand, similar recoveries to the Folch method are reported, but on the other hand, there is a suggestion of thermolabile analytes degradation and thus associated lower recoveries. Another drawback of SE is high solvent consumption, long extraction times (i.e., more than three hours) and the use of toxic solvents [11,50]. Greener extraction alternative represents supercritical fluid extraction (SFE), where the most suitable extraction medium is CO_2_, which is non-toxic and has polarity like pentane in the supercritical state. These facts make SFE very attractive for the extraction of non-polar lipids. If the extraction of polar lipids is needed, it is possible to add an organic modifier to CO_2_, such as EtOH, MeOH or EtOAc [11,21]. In several cases [23,51,52,53], higher efficiency of extraction and recovery of SFE compared to classical extraction methods was confirmed [21]. A comparison of different extraction techniques used in lipid analysis is shown in Table 2. Each extraction method has advantages and disadvantages. The choice of the most suitable one, therefore, depends mainly on the groups of lipids that need to be extracted and on the aims of the analysis.

### 2.2. Derivatization

Chemical modification (derivatization) of lipids represents an additional step in sample pretreatment in lipid analysis. The use of derivatization is essential in GC analysis, primarily because it enables the analysis of analytes, but also provides advantages such as increased selectivity or ionization efficiency. The possibility of using an isotopic labeling (IL) strategy can also be considered as an advantage. IL represents a valuable derivatization concept that can be used in the case of quantitative GC-MS or LC-MS analysis [21,56,57]. This strategy is based on the labeling of standards or control samples with an isotopic derivatization reagent (heavy labeling), while the products of the reaction represent the internal standards (IS) and the sample is labeled with a non-isotopic derivatization reagent (light labeling). After reaction completion, both parts are mixed and analyzed using an appropriate instrumental method [58]. Derivatization is most often applied in GC-MS analysis of FAs [26], but also in the case of analysis of glycerol lipids, sphingolipids, phospholipids or steroids [59]. Even though there are a lot of derivatization methods available for GC-MS analysis of lipids, the use of LC-MS is preferred [26,56]. In recent years, derivatization has been used in several cases, including in LC-MS, especially in short-chain fatty acids analysis [60,61]. An example can be the recent work of Wang et al., who developed highly fluorescent derivatization reagent—1,3,5,7-tetramethyl-8-butyrethylenediamine-difluoroboradiaza-s-indacene (TMBB-EDAN) for determining *trans*-fatty acids in food samples. The method showed good linearity and low detection limits in the range of 0.1–0.2 nM [62]. Despite advantages, derivatization chemically changes the lipid molecule, which can lead to the loss of more information about individual analytes, which, together with the time-consuming nature of derivatization, represents disadvantages [59,63]. In the future, it could be a challenge or motivation not only to synthesize new derivatization reagents but also to speed up or improve existing methods.

## 3. Instrumental Analysis of Lipids

After extraction, lipid analysis using instrumental methods is the next step in the process. For this purpose, nuclear magnetic resonance (NMR) or MS can be used. NMR spectroscopy (i.e., 1H, 13C, 31P) allows the elucidation of lipid structures as well as qualitative and quantitative analysis [21]. For NMR analysis of extracted lipids, it is crucial to dissolve them in an appropriate solvent, such as deuterated MeOH or CHCl_3_, just before the analysis. NMR plays a significant role, particularly in studying membrane lipid profiles or interactions between proteins (or peptides) and lipids. Conversely, analyzing complex matrices without proper extraction can be challenging due to crowded one-dimensional NMR spectra [26,59]. NMR is more often used in metabolomics than in lipidomics. A comprehensive summary, as well as all aspects of the use of NMR in lipidomics, have been reviewed a few years ago by Lia et al. [64].

On the other hand, MS provides the same data on the analyzed samples, but has a higher sensitivity than NMR [21]. Moreover, according to the recently published papers, MS approaches are the dominant ones in lipid analysis [63]. However, the use of MS is much more frequent due to the variety of techniques it offers, whether within the framework of shotgun lipidomics or the possibility of connection with effective separation techniques such as LC or even today less used gas chromatography (GC) or thin-layer chromatography (TLC), which was used in past. In addition, a relatively large number of different ion sources or mass analyzers are commercially available for both identification and quantification or MS lipids imaging [21,56,65]. In lipidomics analysis, liquid chromatography is most often used, as well as direct infusion (DI)-MS [66,67].

### 3.1. Direct Infusion MS

**Figure 4:**
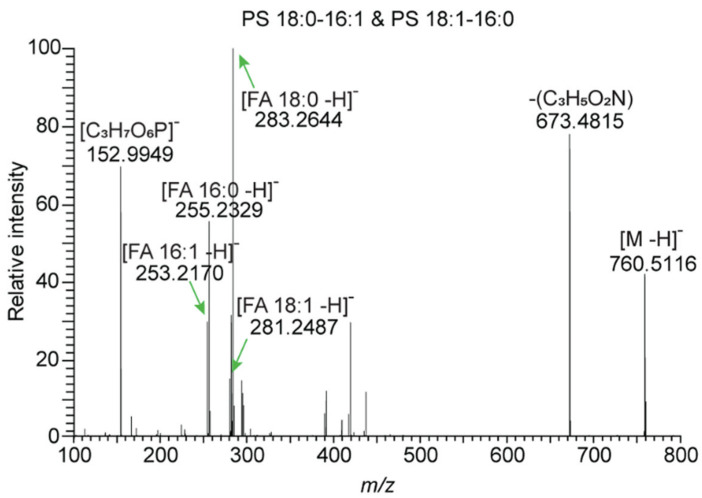
Illustration of FT MS/MS spectrum of PS 34:1 (in negative ionization mode); Adapted with permission from [69]. Copyright 2020 American Chemical Society.

Extracted lipids can be analyzed directly using MS without their previous separation. In the case of lipidomic analyses, this technique is also referred to as shotgun lipidomics [65]. This technique has developed over the years to its present form. It represents a simple but powerful tool for fast, reliable, sensitive, and reproducible lipidomic analyses, while a triple quadrupole (QqQ) or hybrid mass analyzers like Orbitrap, quadrupole-time of flight (QTOF) or Fourier transform ion cyclotron resonance (FT-ICR) can be used as mass analyzers [56,68]. An example of the use of a high-resolution mass spectrometer (HRMS) in shotgun lipidomics is presented in a paper by Nielsen et al. [69]. The authors used a hybrid quadrupole-Orbitrap mass spectrometer with Fourier transformation (FT) equipped with nano-electrospray ionization (nano-ESI) working either in positive or negative mode to the quantitative shotgun lipidomic analysis of the mammalian sample. This approach enabled them to quantify sub-picomole levels in 35 of 38 lipid classes [69]. An illustrational FT MS/MS spectrum of PS 34:1 (in negative ionization mode) is shown in Figure 4.

The FT MS/MS spectrum contains fragments: (C_3_H_5_O_2_N)—neutral loss and [C_3_H_7_O_6_P]^−^, which are major lipid class-specific fragments and fragments: [FA*sn*1−H]^−^ and [FA*sn*2−H]^−^, which represent species-specific acyl chains fragments. The ability to distinguish and identify individual fragments allows the distinction of PS 18:0–16:1 and PS 18:1–16:0 isomers, both of which we could refer to as PS 34:1 [69].

However, the DI-MS-based approach has some limitations. One of them is ion suppression, which could have a negative effect on ion formation, detection, and accuracy of quantification. Ion suppression is a limitation for lipid classes, which are less ionized, or their responses are low and may fuse in the background noise. Another disadvantage that this approach suffers from is the artifacts generated in the ion source, which are present in ESI-MS due to in-source fragmentation. This results in the inability of this approach to distinguish artifactual peaks from lipid peaks in mass spectra. The third issue in DI-MS analysis is the possibility of the overlapping of isomeric or isobaric mass between lipid species, making the identification of lipid isomers unfeasible [70]. Multi-dimensional mass spectrometry-based shotgun lipidomics (MDMS-SL) or differential mobility spectrometry (DMS) was utilized to overcome some of these issues [56]. DMS shotgun lipidomic analysis was recently utilized by Baolong Su et al. (2021) [71]. Authors have also developed a specific application, Shotgun Lipidomics Assistant (SLA), which facilitates DMS-based lipidomics workflows. Using these approaches, the authors were able to analyze more than 1450 lipid species [71]. Other lipid analyses utilizing DI-MS are listed in Table 3.

### 3.2. Mass Spectrometry Imaging

Mass spectrometry imaging (MSI) represents a group of direct MS label-free visualization techniques that do not require sample pretreatment, as needed in other discussed methods [76,77]. In MSI techniques, only a thin slice of sample is required. It is usually attached to a suitable surface and directly analyzed [21]. In conjunction with MSI, soft ionization techniques such as desorption electrospray ionization (DESI), secondary ion MS (SIMS) or matrix assisted laser desorption/ionization (MALDI) are used [78,79]. 

In SIMS, the primary ion beam (Ga, Si or Cs) is accelerated to bombard the surface of the sample and to release secondary ions that can be detected by MS. SIMS has high spatial resolution and thus has the capability of analyzing surfaces of cell or tissues on the molecular level [80,81]. SIMS is most commonly utilized with a TOF analyzer (TOF-SIMS). Despite the fact that applications of SIMS in lipidomics starts much later than MALDI, developments in SIMS such as introduction of nanoSIMS or cluster ion beams (i.e., Bi^3+^ Au^3+^, Au^9+^) that are able to reduce secondary ions fragmentation has led to improved spatial resolution and analytical sensitivity [59,80,81]. Advances in TOF-SIMS were recently reviewed in detail by Jia et al. [82] and the important role of SIMS in lipid imaging was recently proven by Ren et al. [83]. A single-cell lipidomic study was performed using TOF-SIMS analysis of mammalian cells (cardiomyocytes (CMs)). TOF-SIMS surface analyses were performed using primary cluster ion beams Ar_2000_^+^ (for intracellular surfaces) and Bi^3+^ (for cell surfaces and intracellular surfaces images). Thanks to this technique, the authors were able to study lipid metabolism of single cardiomyocyte and identify characteristics associated with heart failure [83].

In MALDI, the surface of matrix-coated sample is irradiated by a laser under vacuum or at atmospheric pressure (AP-MALDI) [76,84]. For good ionization of lipids in MALDI-MSI, an appropriate matrix should be chosen, i.e., 9-aminoacridine (9-AA), 2,5-dihydroxybenzoic acid (DHB) or *N*-(1-naphthyl)ethylenediamine hydrochloride [84]. In addition to MALDI, water-assisted laser desorption ionization (WALDI)-MSI represents an alternative approach where endogenous H_2_O is used as the MALDI matrix [76]. Thanks to the years of improvement in instrumentation and bioinformatics, MALDI-MSI was developed to a method capable of lipid classes and species identification and semi-quantification with no need to use chromatographic separation [76,85]; it is therefore considered as a universal tool for the study of lipids [76]. Evidence of the versatility of MALDI-MSI was recently proven by Martín-Saiz et al. [86]. The authors used a combination of two independent methods, MALDI-MSI and HPLC-MS, for lipids screening in clear cell renal cell carcinoma patients. Analysis of samples using both methods revealed differences between them in terms of the number of detected and identified lipid species (344 by HPLC-MS in ESI-mode and 148 by MALDI-MSI). Moreover, thanks to the spatial resolution of MALDI-MSI, authors were able to get information about studied samples, i.e., the existence of different tumor cell populations or the existence of necrotic areas [86].

Both techniques (SIMS, MALDI) work in a vacuum but MALDI can also operate near or at atmospheric pressure. The non-destructive soft ionization technique (DESI) is the most widely used ambient ionization technique for lipid MSI when working at atmospheric pressure [87,88]. In DESI, desorption of analyte molecules and their ESI ionization is performed in one step. DESI-TOF or even DESI-QqQ setups could be used for analysis. For the highest possible spatial resolution, experimental parameters of DESI, such as solvent (usually MeOH), solvent flow rate, nebulizing gas flow rate (N_2_), capillary and cone voltages and sprayer geometry [87,89], should be optimized. Despite optimizing these parameters, DESI has lower spatial resolution compared to MALDI [87]. On the other hand, sensitivity and spatial resolution could be improved using nanoDESI or a technique called airflow-assisted desorption electrospray ionization (AFADESI). A detailed comparison of MALDI, DESI and AFADESI for MSI was recently published by He et al. [90]. In 2019, Nguyen et al. [91] used nanoDESI for MSI lipid profiling of mouse lung tissues. This method showed comparable coverage of lipids to LC-MS/MS method. Furthermore, the method was able to provide spatial localization (with sufficient spatial resolution) not only of lipids but also small and nonlipid molecules that are not detected in LC-MS/MS lipidomics analysis [91]. All MSI methods used for lipid analyses are shown in Table 4.

### 3.3. Ion Mobility Spectrometry (IMS-MS)

Within lipid analysis, ion mobility (combined with MS: IMS-MS) is a separation technique that separates analyte ions based on their mobilities in an inert gas (nitrogen or helium) using electric field (static or modulated) gradient. Identification (and quantitation) of classes of lipids can be conducted using QqQ working in selective reaction monitoring (SRM) mode. Higher mass resolution analyzers, such as hybrid ion trap-Orbitrap [108] and Q-Orbitrap [109] or Q-TOF [110,111], can work in parallel reaction monitoring (PRM) mode to detect all fragment ions; this allows the identification of lipid species or subspecies [112,113,114]. IMS can be integrated into DI-MS or coupled with chromatographic methods, such as GC or more frequently LC. Incorporating IMS into LC-MS can achieve a new dimension of separation and thereby increase not only selectivity and accuracy but also the sensitivity of the method. Moreover, isomeric/isobaric lipids can be separated; when using MS without IMS, this could not be effectively resolved [85]. Several technologically different variants of IMS-MS are nowadays commercially available: *(i)* trapped ion mobility spectrometry (TIMS), *(ii)* traveling wave ion mobility spectrometry (TWIMS), *(iii)* drift time ion mobility spectrometry (DTIMS), *(iv)* high field asymmetric waveform ion mobility spectrometry (FAIMS), *(v)* Differential ion mobility spectrometry (DIMS) and *(vi)* differential mobility spectrometry (DMS) [85,115].

Furthermore, a bioinformatics approach based on collision cross section (CCS) can also be included in these IMS techniques [114,116]. CCS represents shape-related physical properties of an ion in specific experimental conditions [115,117]. A few years ago, CCS lipid databases were established, in which lipid CCS values are obtained either experimentally (measurement of authentic lipid standards) or theoretically predicted using bioinformatic approach (based on experimentally measured CCS values). Parameters such as retention time, accurate mass, MS/MS spectra or CCS make this a promising tool for improving confidence in lipid identification [118,119]. However, there is still a big challenge due to the limited number of lipids integrated in these CCS databases [85,119]. To cope with this limitation, a relatively new LipidIMMS Analyzer used for the identification and quantification of lipids was introduced. The database contains more than 260,000 lipids; for each lipid, retention time, m/z, MS/MS spectra and CCS parameters are available [85,118]. An overview of other DI-MS approaches utilizing IMS in lipid analysis is shown in Table 5.

### 3.4. LC-MS

Some of the drawbacks mentioned for the direct MS approach may be solved by introducing a separation step: liquid chromatography (LC) before MS detection [125,126]. LC-MS is the most frequently used tandem of analytical techniques used in lipid analysis, especially in lipidomics [21]. Previously, thin-layer chromatography (TLC) or high-performance thin-layer chromatography (HPTLC) were also used, but nowadays, high-performance liquid chromatography (HPLC) or ultra-high-performance liquid chromatography (UHPLC) utilizing capillary or nano-columns are the most commonly used platforms of LC [127,128]. In LC/MS spectra, data can be collected in positive or negative ESI ionization modes, or more rarely polarity switching ionization mode. Due to the high requirements for the quality of analyses, it is necessary to choose a suitable mass analyzer to study the lipids. Nowadays, it is typical to use hybrid mass analyzers such as Orbitrap, QTOF or even combined quadrupole with Orbitrap (Q-Orbitrap). Development of these analyzers greatly improves the identification [127,129]. In the case of quantitative analysis of lipids, use of QqQ is typical [67]. Quantification could be performed using internal standards (IS). Of course, especially in lipidomic analyses, it is not possible to have an IS corresponding to every lipid; as such, commercially available pre-prepared mixes of selected lipids with a precisely defined concentration are used for this purpose [56,130]. 

**Figure 5:**
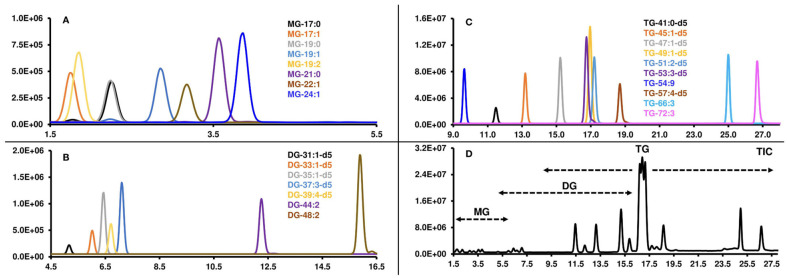
Extracted ion chromatograms (EIC) of selected nonpolar lipid classes: (**A**): MAG; (**B**): DAG; (**C**): TAG separation on RP-C30 column; (**D**): Total ion current (TIC) with display of total number of ions and their distribution on chromatogram. Adapted from [125]. Copyright 2022 Frontiers.

Separations are preferably performed in reversed-phase (RP) mode utilizing a stationary phase with different alkyl chains (C8, C18, C30, etc.), which is suitable for the majority of lipid classes [25,56,127]. Composition of mobile phase is an important factor in lipid analysis. Mixtures of H_2_O and organic solvents like ACN, MeOH or IPA are used with the addition of volatile buffers, i.e., acetic acid, formic acid, ammonium acetate or ammonium formate [127,131]. More demanding quantitation in lipid class separation (different retention times of internal standards and analytes) and inappropriate retention of more polar lipids (like phospholipids) RP stationary phase are the main drawbacks of this strategy [25,131]. On the contrary, an alternative approach is to use a normal-phased (NP) separation system or, more likely, hydrophilic interaction chromatography (HILIC), which can be defined as a subclass of NP system but with the possibility of using mobile phases as in RP mode. HILIC is suitable for the separation of polar lipids and for reliable quantitation because of similar retention times of lipids and internal standards [129]. On the other hand, retention of some lipid classes such as nonpolar (i.e., TAG, CE) or lipids containing one -OH group is poor [127]. Representative example of relevance of RP and HILIC in lipid analysis or in lipidomics was published by Romsdahl et al. [125]. The authors presented targeted lipidomic workflow for polar and nonpolar lipids characterization by two LC-MS methods. The method for determination of nonpolar lipids used the RP-C30 column and offered the possibility to analyze more than two hundred nonpolar lipids. An example of extracted ion LC-MS chromatograms of selected nonpolar lipid classes (MAG, DAG and TAG) separation on the RP-C30 column is shown in Figure 5. The second LC-MS method was based on HILIC using the NH2 column. A total of 260 molecular species from 12 classes of lipids were analyzed using the HILIC method. The use of two separate methods was able to prevent possible peak overlapping, which is undesirable in the quantification process [125]. 

As can be seen from Figure 5, earlier eluted lipid species are those with greater FA unsaturation. The most nonpolar species (TAGs) were eluted in the range from 9 min to 27 min of the chromatogram [125]. Despite certain advantages of HILIC, RP chromatographic system remains dominant. A list of LC-MS and SFC-MS strategies published from 2019 to 2023 is shown in Table 6. 

### 3.5. Supercritical Fluid Chromatography—Mass Spectrometry (SFC-MS)

Another chromatographic approach based on LC principles coupled with MS is supercritical fluid chromatography (SFC-MS) or the more powerful mode of this technique, known as ultrahigh-performance supercritical fluid chromatography-MS (UHPSFC-MS). In SFC, supercritical CO_2_ is used as a mobile phase, where the improved chromatographic performance is the result of a higher diffusion coefficient and lower viscosity of the supercritical mobile phase. The addition of organic modifiers (i.e., MeOH, EtOAC) to the mobile phase creates the possibility of separation of large groups of analytes from highly nonpolar to highly polar [132,133], making this technique suitable for lipid analysis. All aspects of the SFC-MS technique in lipidomic analysis were comprehensively discussed by Wolrab et al. [134]. In 2021, Hayasaka et al. [135] used the SFC-MS method for the analysis of lipids in small extracellular vesicles and cells. Supercritical carbon dioxide was supplemented by 0.1% (*w*/*v*) ammonium acetate in 95% (*v*/*v*) MeOH as a mobile phase. SFC chromatograph was equipped with QqQ mass analyzer with ESI interface. This approach enabled the quantification of five hundred lipids [135]. Despite the advantages of UHPSFC-MS, such as noteworthy sensitivity (especially for non-polar lipids) or its important for clinical applications (high-throughput analysis), the potential of including SFC-MS or UHPSFC-MS techniques into lipidomic studies has not yet been fulfilled. This is primarily due to lack of experience with this technique and also due to low upper pressure limit 400–600 bar (in comparison with UHPLC-MS technique, where upper pressure limit can reach 1300 bar) [134].

### 3.6. Gas Chromatography—Mass Spectrometry (GC-MS)

In GC, an inert carrier gas, used as a mobile phase, carries the analytes through a narrow, long column, where their separation then occurs. A basic condition of GC analysis is sufficient volatility of analytes and their thermal stability [21,119]. Because of this fact, the use of GC in lipid analysis is limited. Lipids that are not volatile or thermally stable must be derivatized and then analyzed by GC [119]. The easiest example of the derivatization procedure is the formation of highly volatile fatty acid methyl esters (FAME) using MeOH as a derivatization reagent. Other derivatization procedures utilizing agents such as heptafluorobutyric acid (HFB) or pentafluorobenzoyl (PFB) have been proposed [136]. However, as mentioned above, derivatization chemically changes the lipid molecule, which is undesirable in some cases depending on the aims of the analysis [21]. 

In combination with MS detection, chemical (CI) or electron ionization (EI) are used because of advantages such as high sensitivity, high resolution and compound libraries for identification. Even though GC-MS is not suitable for large-scale lipidomic studies, it can be used advantageously for the analysis of sterols, FAs and *cis*/*trans* isomers [119,137].

**Table 6:** Overview on lipid analysis in different matrices using LC-MS and SFC-MS approach.

Sample	Analytes	Extraction Type	Extraction Solvent	Method	Approach	Results	Ref.
***Biological samples***							
rat serum, brain tissue	SP	LLE	CHCl_3_:MeOH (9:1)	RPLC-MS/MS	targeted	method for quantification of SP in biological samples	[138]
human plasma, mouse serum	lipidomic profiling	BUME	BuOH:MeOH (1:1)	LC-MS/MS	untargeted	88 lipid species were identified as significantly different between wild type CerS2 null mice	[139]
human serum	lipid profiling	LLE	CHCl_3:_MeOH (3:1)	UHPLC-HRMS	untargeted	potentially 12 lipids can serve as diagnostic markers of colorectal adenoma	[140]
serum	HDL	LLE (Folch method)	CHCl_3:_MeOH	LC-MS/MS	targeted	association of MetS with impairment of phospholipid metabolism in HDL, with obesity and insulin resistance	[141]
plasma	SP	OPE	MeOH	LC-MS/MS	targeted	33 identified SP	[142]
mouse tissue	lipid profiling	OPE	MeOH:H_2_O (80:20)	LC-MS/MS	-	identification of major cardiolipin molecular species by BRI-DIA and hybrid methods	[143]
rat serum	lipid markers of CHD	LLE	MTBE	UPLC-HDMS	-	GP and SP metabolism as targets for the treatment of CHD	[144]
porcine brain extract	lipidomic profile	LLE	MTBE	RP-LC-MS	-	development of microgradient fractionation of total lipid extract for lipidomic analysis.	[145]
renal biopsies	lipid biomarkers of Fabry disease	LLE (Folch method)	CHCl_3:_MeOH	UHPLC-HRMS	untargeted	identification of biomarkers of Fabry disease	[146]
pancreatic cancer cells, extracellular vesicles	lipids and metabolites	LLE	CHCl_3:_MeOH	SFC-MS	-	identification of 494 lipids	[135]
human serum	PCs	SPE	eluted with IPA	LC-MS/MS	-	elevation of oxidized PCs in the acute phase of KD	[147]
human cancer cells and EVs	lipidomic profile	LLE (Bligh and Dyer method)	CHCl_3:_MeOH	SFC-MS	-	breast cancer EVs selectively loaded with lipids supporting tumor progression	[148]
human plasma	polar lipids	OPE	MeOH	LC-MS/MS		method development for monitoring of 398 polar lipids	[149]
plasma, urine	oxidation products of PUFA	LLE (Folch method)	CHCl_3:_MeOH	LC-QTOF-MS/MS	targeted	method development for measuring of oxidation products of PUFA	[150]
human CSF	VLCFA	SPE, LLE + derivatization	octane:EtOH (88:12) + DAABD-AE	UPLC-MS/MS	targeted	assay development for measuring of VLCFA biomarkers	[151]
human plasma	lipidome	LLE, UAE	CHCl_3:_MeOH (3:1)	UHPLC-MS	targeted, untargeted	PC (18:1/P-16:0), PC (o-22:3/22:3), PC(P-18:1/16:1) as biomarkers of metabolic syndrome	[152]
human plasma	lipidomic biomarkers	OPE	IPA	LC-MS	targeted	reference for bladder cancer and renal cell carcinoma biomarker discovery	[153]
human fibroblasts	unsaturated FA	LLE	MTBE	LC-MS	targeted	complete characterization of FA species	[154]
mouse plasma	CE, FA, PC, NAE, SM	LLE (Folch method)	CHCl_3:_MeOH	UHPLC-HR-MS	untargeted	identification of plasma lipid species associated with pain and/or pathology in a DMM model of OA	[155]
human plasma	LPCs	OPE, UAE	MeOH:ACN	LC-ESI-MS/MS	targeted	identification of 60 LPCs	[156]
human plasma	lipidomic screening	LLE (Bligh and Dyer method)	CH_3_OH–CH_2_Cl_2_	UPLC-MS	untargeted	increasing of TAGs levels of advanced-stage CRC patients compared with early-stage CRC patients	[157]
human serum	LPC, PC, LPE, PE, LPS, PS, LPG, PG, LPI, PI, LPA, PA, SM, MAG, DAG, TAG, CL, Cer, CE	LLE (Folch method)	CHCl_3:_MeOH (2:1, *v*/*v*)	RPLC-MS/MS	untargeted	identification of 753 lipids	[158]
mouse tissues and fluids	acylcarnitines	OPE + derivatization	MeOH:H_2_O + 3-NPH	LC-MS	targeted	identification of 123 acylcarnitines	[159]
plasma, fecal	SCFAs	OPE + derivatization	H_2_O + 2- bromoacetophenone	LC-MS/MS	targeted	identification of 7 SCFAs	[160]
plasma, tissue	lipid mediators	SPE	eluted with methyl formate	LC-MS/MS	untargeted	novel tool for studying complete profile of lipid mediators in biological samples	[161]
human serum	lysosphingomyelin-509	OPE	EtOH:H_2_O (3:1, *v/v*)	LC-MS	targeted	identification of lysosphingomyelin-509	[162]
mouse liver	lipid profile	LLE	MeOH:DCM (1:3)	UPLC-MS	-	significant differences in lipid profiles of SCID and chimeric PXB liver-humanized mice	[163]
***Food***							
green, red lettuce	sulfolipids, galactolipids	LLE (Folch method)	CHCl_3:_MeOH (3:2)	LC-ESI-MS/MS	targeted	oxidized SQDG as potential markers for abiotic stress factors	[164]
geopropolis	lipid profiles	LLE	MeOH, CHCl_3_	LC-HRMS	-	identification of 61 lipids	[165]
oil palm	lipid profiles	LLE	MTBE	LC-MS	targeted	lipidomic tools for analysis of lipid composition variability in oil from palm	[166]
fish oil, mushroom extract	FuFA-containing TAGs	LLE, UAE	cyclohexane:EtOAc (46:54)IPA:n-hexane (1:4)	LC-HRMS	-	identification of 39 different FuFA-containing TAGs	[167]
olive fruit seeds	polar lipids	LLE (Folch method)	CHCl_3:_MeOH (2:1)	HILIC-HR-MS/MS	untargeted	identification of 94 lipids	[168]
coffee	specific lipids of interest for each coffee origin	LLE	MTBE	LC-MS/MS	targeted	determination of coffee origin based on its lipid profile	[169]
donkey meat	lipid profiles	LLE (Folch method)	CHCl_3:_MeOH (2:1)	LC-MS	untargeted	identification of 1143 lipids	[170]
milk	HFAs	OPE	MeOH	LC-HRMS	-	quantification of 19 free HFAs	[171]
extra virgin olive oil	FFAs, FFA methyl- and ethylesters, MAGs, triterpenoids, TAGs	OPE	IPA	LC-MS/MS	-	potent tool for studying variability of lipid species in olive oil	[172]
potatoes	polar lipids	LLE (Bligh and Dyer, Folch, ”Green” Folch, Matyash, extraction with n-hexane)	CHCl_3:_MeOH EtOAc:MeOH MTBE *n*-hexane	UPLC-MS	targeted, untargeted	“Green” Folch method (with EtOAc)—the most suitable extraction method	[173]
**Pharmaceuticals**							
dietary supplements	lipid profiling	-	-	LC-MS	-	production of different lipid classes by different based ingredients products	[174]
**Bacteria**							
*Pseudomonas aeruginosa*	phospholipids	LLE (Bligh and Dyer)	CHCl_3:_MeOH	LC-MS/MS	-	the growth medium can influence membrane lipid composition	[175]
*C. eiseniae, Olivibacter* sp.	glycerophosholipids	LLE	MTBE MeOH	UHPLC-HR-MS	-	identification of 2 novel glycerophospholipids, 2 novel LAAs	[176]
*Escherichia coli*	GPs	LLE	MTBE	UPLC-MS/MS	targeted	transferability of method to any UPLC-MS/MS system with no hardware modification need	[177]
**Fungi**							
marine fungi	ergosterol	LLE (Bligh and Dyer)	CHCl_3:_MeOH	LC-MS/MS	targeted	highly sensitive method for measuring fungal biomass	[178]
**Plants**							
plant tissue	polar and non-polar lipids	LLE	different solvents optimization of extraction	UHPLC-MS/MS	-	method development for evaluating of polar and non-polar lipids	[125]
tobacco hairy roots	GPL	LLE (Bligh and Dyer)	CHF_3:_MeOH	HILIC-MS/MS	targeted	method development for simultaneous determination of different phospholipids	[179]
*Arabidopsis thaliana*	lipid profiling	LLE	CHCl_3:_MeOH:H_2_O (1:2.5:1) MeOH:MTBE (1:3) IPA + CHCl_3:_MeOH:H_2_O (30:41.5:3.5) IPA + CHCl_3:_H_2_O (5:2) + CHCl_3_:MeOH (2:1)	LC-MS	targeted, untargeted	single-step extraction method for untargeted lipidomic analysis	[34]

3-NPH: 3-nitrophenylhydrazine; CHD: coronary heart disease; CL: cardiolipin; CRC: colorectal cancer; CSF: cerebrospinal fluid; DAABD-AE: (4-[2-(*N*,*N*-dimethylamino)ethylaminosulfonyl]-7-(2-aminoethylamino)-2,1,3-benzoxadiazole]; DCM: Dichloromethane; DMM: destabilisation of the medial meniscus; EVs: extracellular vesicles; FFAs: free fatty acids; FuFA: furan fatty acids; GPL: Glycerophospholipids; HDL: high-density lipoprotein; HFAs: hydroxy fatty acids; KD: Kawasaki disease; LAAs: lipoamino acids; LPA: lysophosphatidic acid; LPC: lysophosphatidylcholine; LPE: lysophosphatidylethanolamine; LPG: lysophosphatidylglycerol; LPI: lysophosphatidylinositol; MetS: metabolic syndrome; NAE: *N*-acylethanolamines; OA: osteoarthritis; PUFA: polyunsaturated fatty acids; SCFAs: short-chain fatty acids; SQDG: sulfoquinovosyl diacylglycerols; UPLC/UHPLC: ultra high-performace liquid chromatography; VLCFA: very long chain fatty acids.

## 4. Data Analysis

Computational analysis of lipidomic data typically comprises three parts: processing of raw data, statistical analysis and enrichment analysis/visualization. 

As mentioned in the introduction, two approaches are commonly used in lipid analysis: targeted and untargeted. The processing of raw data significantly differs between these two approaches. Identifying lipids after analysis is challenging with the untargeted approach. In the case of LC-MS analysis, the Metabolomics Standards Initiative (MSI) proposed that a minimum of two different types of data are needed for molecule identification, for example fragmentation MS spectrum and retention time [180]. In 2022, the Lipidomics Minimal Reporting Checklist was introduced to unify the minimal requirements for generating, reporting and publishing lipidomic data [181].

At this point, it should be mentioned that a very important step preceding data analysis is data acquisition. To acquire MS/MS data, two main acquisition techniques, data-dependent acquisition (DDA) and data-independent acquisition (DIA), are used [1,180]. The main difference between these two approaches is in the number of compounds for which the MS/MS spectrum is acquired. In the DDA technique, a greater number of possible MS/MS fragmentation spectra can be obtained for fewer compounds in a single run. On the other hand, MS spectra are purer due to the narrow mass isolation window [180,182]. However, the incapability of producing MS/MS fragments of each precursor ion found in spectra (particularly for low-abundance precursor ions) and false MS/MS fragment contamination because of precursor window width are disadvantages of DDA [1]. In a single injection run using the DIA technique, possible MS/MS fragmentation spectra can be obtained for all compounds [180]. DIA could be performed in two ways: (*i)* all-ion fragmentation (MS^ALL^ and MS^E^) or (*ii)* all theoretical fragment-ion spectra sequential window acquisition [1,183]. Due to the structural similarity of compounds, MS/MS spectra could be complicated and lead to incorrect data interpretation [180].

A routinely used acquisition method in lipidomics is DDA [1,143,184], not only because of the high quality of spectra but also as is little or no spectra processing before their usage [184]. On the other hand, novel workflows for the DIA acquisition method have been recently developed [139,143,163]. As an example of such approach is the work of Duan et al. [143]. Based on LIPID MAPS and MS DIAL 4 (lipidome atlas), authors created an ion list consisting of biologically relevant lipids. After extraction of lipids from mouse tissues, LC-MS/MS analysis was conducted using DDA, BRI-DIA (biologically relevant ions-DIA) and hybrid mode (BRI-DIA followed by DDA) approaches. It is important to mention here, however, that while hybridizing DIA and DDA modes are trending in metabolomics or proteomics, they have not adapted well in HRMS lipidomics. In addition to other results from this study, the authors concluded that DIA was comparable to DDA, and, moreover, that DIA was better in terms of lipid identification consistency [143].

In the past few years, many bioinformatic tools have been created to evaluate data obtained from untargeted lipid analysis and their subsequent statistical processing for the proper interpretation of the results. The choice of evaluation software often depends on the equipment used, e.g., evaluation software Analyst or MultiQuant (Sciex); MassLynx MS and Progenesis QI (Waters); Masshunter (Agilent) or LipidSearch (Thermo). On the other hand, it is possible to use other freely available software or internet modules for MS data processing, e.g., Skyline, MSDial, MZmine, LipidMatch and others [127]. Zeng et al. provided an example of utilizing such software for the determination of sphingolipid content. [138]. The authors used Masshunter Workstation after LC-MS/MS analysis of rat serum, brain tissue and HT22 cells. This evaluating tool helped to determine several sphingolipids changes in these matrices [138]. Another example is the use of Analyst software (Sciex), which was used by Aurum et al. [169] to evaluate LC-MS/MS lipid profiles obtain by analysis of two types of Indonesian coffee. Subsequent statistical processing of obtained data was carried out in MarkerView software. Thanks to LC-MS/MS analysis and combination of software, the authors were able to identify 85 lipid species from 5 different lipid classes [169]. Another group of authors, Kirkwood et al. [185], made a protocol for utilizing Skyline software for processing and annotating multidimensional lipidomic data and described each step of this processing in detail. Moreover, a lipids library containing more than five hundred lipids was created [185]. Zhou et al. [118] developed a software tool, LipidIMMS Analyzer, for more accurate identification of lipids analyzed by IM-MS. For each lipid, the software comprises four-dimensional (4D) structural information (retention time, m/z, MS/MS spectra and CCS). Moreover, the software contains a database with more than 260,000 lipids. In addition, the authors also proposed a complete workflow for LipidIMMS Analyzer [118].

Each of these software tools has its advantages and limitations, particularly in terms of being able to evaluate only data from specific acquisition modes, or limited output formats (.csv, .xlsx, .html) that only certain statistical software can process [112]. 

In targeted mode, the primary focus is on manually verifying the correct peak selection, and integration using retention time patterns. This is typically done using vendor-specific software.

Processed data, usually in .csv or .txt format, are subjected to data transformation, normalization, subsequent statistical analysis, and enrichment/pathway analysis. This process is similar to that in metabolomic analysis and involves the use of similar tools. The most frequently used tools include MetaboAnalyst [168], and specific packages for metabolomics/lipidomics written in Python [186], R [187], or Matlab [188]. Delving into detailed discussions of specific packages and computational tools exceeds the scope of this manuscript, focusing instead on the analytical chemistry approaches in lipidomics. For in-depth exploration, readers are encouraged to consult specialized resources.

Complexity of lipids, difficulties in identifying lipid isomers or challenging lipids quantification create limitations in lipid analysis. Moreover, often complicated sample preparation step, which can negatively affect analysis results (especially in term of reproducibility), have to be involved in the process. To overcome these challenges, advancements in mass spectrometry resolution, real-time imaging technologies are needed hand in hand with standardized analytical methodologies in lipid analysis should be established. Additionally, in terms of broader biological context of lipid importance (i.e., physiological, or pathological processes), the use of multi-omics approaches can be essential. Last but not least, advancements in sample pretreatment are needed. Development in miniaturized and automated extraction techniques can lead to increased efficiency of extraction and thus to high quality data obtained from lipid analysis.

## 5. Conclusions

This review provides a comprehensive overview of the analytical methodologies used in lipid analysis in various matrices. Across the available literature, biological matrices (such as plasma, CSF, and urine) are nowadays the most commonly analyzed. Within the framework of lipid analysis, much attention is currently being paid to lipidomics. However, the analysis itself is preceded by sample treatment, where the most common step is the extraction of lipids due to the complexity of matrices. Classical LLE-based extraction methods, such as Bligh and Dyer or Folch, are still being improved and there is an ongoing effort to replace toxic solvents with green ones such as CPME. However, the use of green solvents for extraction is still limited by economic considerations. Other promising extraction methods are SPE, SAP, or SFE, which have not yet reached their potential in lipid analysis. Due to the fact that high-throughput analyses are usually required, MS-based methods are the most used in this field. After considering a lot of factors (i.e., aims of analysis, type of analysis etc.) MS can be used for lipid analysis directly without prior separation by DI-MS (or shotgun lipidomics). However, in most cases, it is essential to include a separation technique before MS, while the most frequently used combination is LC-MS, which represents the combination of two high-performance analytical techniques. The combination of MS with other separation techniques such as GC or CE is less common due to its limitations. However, SFC, which can separate a wide range of lipids, is also gaining awareness. Technological advances in MS techniques caused an increase in the so-called spatial lipidomics, which is ensured by MS imaging techniques. Due to revealing information about connection between lipid changes in the organism and different diseases, there is still emerging interest for introducing novel extraction methods or development of high-precision and high-sensitivity methods for reliable identification and quantification of lipids in the future.

## Figures and Tables

**Table 1:** Lipid classes; adapted from [9,10].

Lipid Class	Example
Fatty acyls	HFAs, FAs
Glycerolipids	MAG, DAG, TAG
Glycerophospholipids	PA, PC, PE, PS
Sphingolipids	Cer, PSL, SM
Sterol lipids	CE, Chol, CS
Prenol lipids	quinine, polyprenol, isoprenoid
Saccharolipids	lipid A
Polyketides	lovastatin

CE: cholesteryl ester; Cer: ceramide; CS: cholesteryl sulfate; DAG: diacylglycerol; FAs: fatty acids; HFAs: hydroxyl-fatty acids; MAG: monoacylglycerol; PAs: phosphatidic acids; PS: phosphatidylserine; PSL: phosphosphingolipids; SM: sphingomyelin.

**Table 2:** Comparison of different extraction techniques used in lipid analysis.

Extraction Method	Advantages	Disadvantages	Ref.
**OPE**	easy to perform possibility of automation low cost precipitation of proteins and insoluble organic species	may not remove interferences efficiently long centrifugation needed	[26,54]
**LLE**	well-established protocols many combinations of solvents could be used (in different ratios) low cost	time-consuming difficult to automate repeated extractions needed challenging organic phase layer transfer	[54]
**SPE**	reduction of matrix effect purification of samples wide range of commercially available SPE sorbents	particularly suitable for targeted analysis long optimization of washing and elution solvents	[27]
**SPME**	requires very small amount of sample reduction of matrix effect small amounts of solvents needed fast extraction	suitable mainly for GC lower extraction ability	[7,27,55]
**UAE**	highly-reproducible time-efficient improve the extraction efficiency in combination with LLE	use of toxic solvents longer extraction times increasing the temperature (because of fictions) which leads to degradation possibly damage hearing	[21]
**MAE**	improvement of extraction efficiency reduction of time and organic solvents consumption	potential degradation of thermally instable lipids long optimization of extraction parameters	[21]
**SE**	provides a high yield of lipids possibility of use with green solvents	continuous heating at the boiling temperature could lead to lipid oxidation and degradation of heat liable, time consuming	[11]
**SFE(SCO_2_)**	shorter extraction times suitable for neutral, low-polarity lipids supercritical CO_2_ is green solvent	extraction of polar lipids requires use of organic modifier high initial costs of equipment	[11,26,42]

**Table 3:** Overview on lipid analysis in different matrices using direct infusion-mass spectrometry (DI-MS).

Sample	Analytes	Extraction Type	Extraction Solvent	Results	Ref.
**rat brain tissue**	lipid profile	OPE	MeOH	direct infusion probe development for metabolomics	[72]
**20 mammalian cells**	19 lipid subclasses	LLE	CHCl_3:_MeOH:IPA, (1:2:4)	determination of different lipid species with potential for clinical applications	[73]
**fermented vegetable juices**	lipid profiling	LLE	MTBE	fermented juices contain more beneficial metabolites and carotenoids than commercial non-fermented juices	[74]
**mammalian samples**	lipidome	LLE (Bligh and Dyer)	CHCl_3:_MeOH	guideline for setting up and using platform for exploring mammalian lipidome	[69]
**bovine milk**	TAG	LLE	CHCl_3_	identification of more than 100 TAGs	[75]

**Table 4:** Overview on MSI methods used for lipid analysis in different matrices.

MALDI-MSI				
Sample	Analytes	Matrix	Results	Ref.
human kidney tissues	lipidome	DAN	comparing of LIMS and HPLC-MS—identification of larger number of species with using HPLC-MS	[86]
rat brain tissue	lipidomic profiles	norharmane	lipidomic spectra showed high consistency between MALDI and WALDI	[76]
human and murine tissue	lipid profiling	DHB	identification of several atherosclerosis- specific lipid biomarkers	[92]
salivary gland tumor tissue	lipidomic profile	DHB	MALDI-MSI complementary diagnostic tool	[77]
human tissue	lipid profiling	DHB	plaque features and specific lipid classes clear colocalization	[93]
osteoarthritic synovial membrane	lipidomic profile	norharmane	novel insight into lipid profiling of synovial membrane	[94]
colorectal cancer tissue	lipidomic profile	9-AA	tool for subtyping the diverse immune environments in CRC	[95]
tumor spheroids	lipid metabolites	DHB	method for detailed information about spheroids and drug relationship	[96]
human tissue	lipid profiling	DHB	diacylglycerols are more abundant in thrombotic area in comparison with other plaque areas	[97]
human kidney tissue	Lipid storage	DHB	sections stored at RT (one week of storage)—largest amount of lipid degradation in comparison with sections stored under N_2_ at −80 °C	[98]
**SIMS-MSI**				
**Sample**	**Analytes**	**Primary Ion Beam**	**Results**	**Ref.**
mammalian CMs	lipid profiling	Ar_2000_^+^ Bi^3+^	identifying of heart failure associated lipids	[83]
lipid extracts, cells, mouse brain tissue	lipid profiling	(CO_2_)_n_^+^ (H_2_O)_n_^+^, (H_2_O)_n_ ^+^(CO_2_)	imaging of LPC for the first time using TOF-SIMS	[99]
*Gammarus fossarum*	lipidome characterization	Bi^3+^ cluster ions	compositional and spatial information of lipids	[100]
infarcted mouse heart tissue	spatial distribution of lipids	gas cluster ion beam (Ar_4000_^+^)	different spatial lipids distributions; insights changes in lipid metabolism following infarction	[101]
**DESI-MSI**				
**Sample**	**Analytes**	**Solvent System,** **Technique**	**Results**	**Ref.**
mice liver tissue	lipid distribution	MeOH:H_2_O (98:2) DESI	zone-specific hepatic lipid distribution of three zones	[102]
human carotid plaque	lipid signatures	MeOH:H_2_O (98:2) DESI	identified lipid species present in plaque (compared with plasma)	[103]
asiatic toad	lipid composition	MeOH:H_2_O (95:5) DESI	significant lipid metabolism changes due to body remodeling during metamorphosis	[104]
xenograft glioblastoma tumour	lipid profiling	MeOH:H_2_O (95:5) 3D DESI	heterogeneous lipid expression is important to aid β-oxidation in hypoxic areas glioblastoma	[105]
cow, sow, mouse ovaries	lipid distribution	DMF:ACN (1:1) DESI	similar lipid signatures of corpora lutea, follicular wall, ovarian stroma independent of the species	[106]
swine fetuses	lipid distribution	DMF:ACN (1:1) DESI	organ-dependent localization of lipids, indication of key lipids related to physiological organogenesis	[107]
mouse lung tissues	lipid coverage	MeOH:H_2_O (9:1) nanoDESI	spatial localization of lipids in tissues. 50% of lipid coverage in comparison with Folch extraction-LC-MS/MS method	[91]

DAN: 1,5-diaminonaphtalene; RT: Room temperature.

**Table 5:** Overview on lipid analysis in different matrices analyzed by IMS.

Sample	Analytes	Extraction Type	Extraction Solvent	Method	Results	Ref.
porcine oocyte	lipidomic profile	LLE	MeOH:CHCl_3_	nanoLC-TIMS-MS	oocyte lipids identification and relative quantification at the single-cell level	[120]
human plasma, serum	lipid profiling	LLE	MTBE:MeOH (10:3)	UHPLC-TIMS-PASEF-MS	Annotation of 370 lipids in reference plasma and 364 lipids in serum sample	[121]
mouse brain tissue	lipid localization	-	MeOH:H_2_O (9:1) nanoDESI	nanoDESI-TIMS-MSI	separation of lipid isomers and isobars and localization in brain tissue	[122]
plasma	lipid profile	LLE	MTBE	UHPLC-TIMS-MS	approach development for untargeted lipidomics	[123]
human plasma, mouse liver, HeLa cells	lipidomic profile	LLE	MeOH:MTBE:H_2_O	nanoLC-TIMS-PASEF-MS	1108 lipids (0.05 μL plasma), 976 lipids (10 μg liver tissue) and 1351 lipids (~2000 HeLa cells) were identified	[124]

PASEF: Parallel accumulation–serial fragmentation.

## Data Availability

Data are contained within the article.

## References

[1] Züllig T., Trötzmüller M., Köfeler H.C. (2020). Lipidomics from Sample Preparation to Data Analysis: A Primer. Anal. Bioanal. Chem..

[2] Xu T., Hu C., Xuan Q., Xu G. (2020). Recent Advances in Analytical Strategies for Mass Spectrometry-Based Lipidomics. Anal. Chim. Acta.

[3] Natesan V., Kim S.J. (2021). Lipid Metabolism, Disorders and Therapeutic Drugs—Review. Biomol. Ther..

[4] Bai L., Bu F., Li X., Zhang S., Min L. (2023). Mass Spectrometry-Based Extracellular Vesicle Micromolecule Detection in Cancer Biomarker Discovery: An Overview of Metabolomics and Lipidomics. View.

[5] Han X. (2016). Lipidomics for Studying Metabolism. Nat. Rev. Endocrinol..

[6] Rod-In W., Monmai C., Shin I., You S.G., Park W.J. (2020). Neutral Lipids, Glycolipids, and Phospholipids, Isolated from Sandfish (*Arctoscopus japonicus*) Eggs, Exhibit Anti-Inflammatory Activity in LPS-Stimulated RAW264.7 Cells through NF-κB and MAPKs Pathways. Mar. Drugs.

[7] Song Y., Cai C., Song Y., Sun X., Liu B., Xue P., Zhu M., Chai W., Wang Y., Wang C. (2022). A Comprehensive Review of Lipidomics and Its Application to Assess Food Obtained from Farm Animals. Food Sci. Anim. Resour..

[8] Lee H.C., Yokomizo T. (2018). Applications of Mass Spectrometry-Based Targeted and Non-Targeted Lipidomics. Biochem. Biophys. Res. Commun..

[9] Kao Y.C., Ho P.C., Tu Y.K., Jou I.M., Tsai K.J. (2020). Lipids and Alzheimer’s Disease. Int. J. Mol. Sci..

[10] Shamim A., Mahmood T., Ahsan F., Kumar A., Bagga P. (2018). Lipids: An Insight into the Neurodegenerative Disorders. Clin. Nutr. Exp..

[11] Saini R.K., Prasad P., Shang X., Keum Y.S. (2021). Advances in Lipid Extraction Methods—A Review. Int. J. Mol. Sci..

[12] Ulmer C.Z., Koelmel J.P., Jones C.M., Garrett T.J., Aristizabal-Henao J.J., Vesper H.W., Bowden J.A. (2021). A Review of Efforts to Improve Lipid Stability during Sample Preparation and Standardization Efforts to Ensure Accuracy in the Reporting of Lipid Measurements. Lipids.

[13] Zhao X.E., Zhu S., Liu H. (2020). Recent Progresses of Derivatization Approaches in the Targeted Lipidomics Analysis by Mass Spectrometry. J. Sep. Sci..

[14] Lange M., Ni Z., Criscuolo A., Fedorova M. (2019). Liquid Chromatography Techniques in Lipidomics Research. Chromatographia.

[15] Li L., Han J., Wang Z., Liu J., Wei J., Xiong S., Zhao Z. (2014). Mass Spectrometry Methodology in Lipid Analysis. Int. J. Mol. Sci..

[16] Belhaj M.R., Lawler N.G., Hoffman N.J. (2021). Metabolomics and Lipidomics: Expanding the Molecular Landscape of Exercise Biology. Metabolites.

[17] O’Donnell V.B., Ekroos K., Liebisch G., Wakelam M. (2020). Lipidomics: Current State of the Art in a Fast Moving Field. Wiley Interdiscip. Rev. Syst. Biol. Med..

[18] Long N.P., Park S., Anh N.H., Kim S.J., Kim H.M., Yoon S.J., Lim J., Kwon S.W. (2020). Advances in Liquid Chromatography–Mass Spectrometry-Based Lipidomics: A Look Ahead. J. Anal. Test..

[19] Köfeler H.C., Ahrends R., Baker E.S., Ekroos K., Han X., Hoffmann N., Holcapek M., Wenk M.R., Liebisch G. (2021). Recommendations for Good Practice in MS-Based Lipidomics. J. Lipid Res..

[20] Howlader M.S., French W.T., Alam M.A., Xu J.L., Wang Z. (2020). Pretreatment and Lipid Extraction from Wet Microalgae: Challenges, Potential, and Application for Industrial-Scale Application. Microalgae Biotechnology for Food, Health and High Value Products.

[21] Teo C.C., Chong W.P.K., Tan E., Basri N.B., Low Z.J., Ho Y.S. (2015). Advances in Sample Preparation and Analytical Techniques for Lipidomics Study of Clinical Samples. TrAC Trends Anal. Chem..

[22] Patel A., Mikes F., Matsakas L. (2018). An Overview of Current Pretreatment Methods Used to Improve Lipid Extraction from Oleaginous Microorganisms. Molecules.

[23] Elst K., Maesen M., Jacobs G., Bastiaens L., Voorspoels S., Servaes K. (2018). Supercritical CO_2_ Extraction of Nannochloropsis Sp.: A Lipidomic Study on the Influence of Pretreatment on Yield and Composition. Molecules.

[24] Lee S.Y., Cho J.M., Chang Y.K., Oh Y.K. (2017). Cell Disruption and Lipid Extraction for Microalgal Biorefineries: A Review. Bioresour. Technol..

[25] Pati S., Nie B., Arnold R.D., Cummings B.S. (2016). Extraction, Chromatographic and Mass Spectrometric Methods for Lipid Analysis. Biomed. Chromatogr..

[26] Jurowski K., Kochan K., Walczak J., Barańska M., Piekoszewski W., Buszewski B. (2017). Comprehensive Review of Trends and Analytical Strategies Applied for Biological Samples Preparation and Storage in Modern Medical Lipidomics: State of the Art. TrAC Trends Anal. Chem..

[27] Liakh I., Sledzinski T., Kaska L., Mozolewska P., Mika A. (2020). Sample Preparation Methods for Lipidomics Approaches Used in Studies of Obesity. Molecules.

[28] Löfgren L., Forsberg G.B., Ståhlman M. (2016). The BUME Method: A New Rapid and Simple Chloroform-Free Method for Total Lipid Extraction of Animal Tissue. Sci. Rep..

[29] Alshehry Z.H., Barlow C.K., Weir J.M., Zhou Y., McConville M.J., Meikle P.J. (2015). An Efficient Single Phase Method for the Extraction of Plasma Lipids. Metabolites.

[30] Liebisch G., Höring M., Stieglmeier C., Schnabel K., Hallmark T., Ekroos K., Burkhardt R. (2022). Benchmarking One-Phase Lipid Extractions for Plasma Lipidomics. Anal. Chem..

[31] Bögl T., Mlynek F., Himmelsbach M., Buchberger W. (2022). Comparison of One-Phase and Two-Phase Extraction Methods for Porcine Tissue Lipidomics Applying a Fast and Reliable Tentative Annotation Workflow. Talanta.

[32] Folch J., Lees M., Sloane Stanley G.H. (1957). A Simple Method for the Isolation and Purification of Total Lipides from Animal Tissues. J. Biol. Chem..

[33] Bligh E.G., Dyer W.J. (1959). The National Research Council of Canada a Rapid Method of Total Lipid Extraction and Purification. Can. J. Biochem. Physiol..

[34] Kehelpannala C., Rupasinghe T.W.T., Hennessy T., Bradley D., Ebert B., Roessner U. (2020). A Comprehensive Comparison of Four Methods for Extracting Lipids from Arabidopsis Tissues. Plant Methods.

[35] Breil C., Abert Vian M., Zemb T., Kunz W., Chemat F. (2017). “Bligh and Dyer” and Folch Methods for Solid–Liquid–Liquid Extraction of Lipids from Microorganisms. Comprehension of Solvatation Mechanisms and towards Substitution with Alternative Solvents. Int. J. Mol. Sci..

[36] Omar A.M., Zhang Q. (2023). Evaluation of Lipid Extraction Protocols for Untargeted Analysis of Mouse Tissue Lipidome. Metabolites.

[37] Lin J.H., Liu L.Y., Yang M.H., Lee M.H. (2004). Ethyl Acetate/Ethyl Alcohol Mixtures as an Alternative to Folch. J. Agric. Food Chem..

[38] Matyash V., Liebisch G., Kurzchalia T.V., Shevchenko A., Schwudke D. (2008). Lipid Extraction by Methyl-Tert-Butyl Ether for High-Throughput Lipidomics. J. Lipid Res..

[39] Aldana J., Romero-otero A., Cala M.P. (2020). Exploring the Lipidome: Current Lipid Extraction Techniques for Mass Spectrometry Analysis. Metabolites.

[40] Wong M.W.K., Braidy N., Pickford R., Sachdev P.S., Poljak A. (2019). Comparison of Single Phase and Biphasic Extraction Protocols for Lipidomic Studies Using Human Plasma. Front. Neurol..

[41] Vale G., Martin S.A., Mitsche M.A., Thompson B.M., Eckert K.M., McDonald J.G. (2019). Three-Phase Liquid Extraction: A Simple and Fast Method for Lipidomic Workflows. J. Lipid Res..

[42] de Jesus S.S., Filho R.M. (2020). Recent Advances in Lipid Extraction Using Green Solvents. Renew. Sust. Energ. Rev..

[43] Probst K.V., Wales M.D., Rezac M.E., Vadlani P.V. (2017). Evaluation of green solvents: Oil extraction from oleaginous yeast Lipomyces starkeyi using cyclopentyl methyl ether (CPME). Biotechnol. Prog..

[44] de Jesus S.S., Ferreira G.F., Moreira L.S., Wolf Maciel M.R., Maciel Filho R. (2019). Comparison of Several Methods for Effective Lipid Extraction from Wet Microalgae Using Green Solvents. Renew. Energy.

[45] Bang G., Kim Y.H., Yoon J., Yu Y.J., Chung S., Kim J.A. (2017). On-chip lipid extraction using superabsorbent polymers for mass spectrometry. Anal. Chem..

[46] Choi J.H., Bang G., Kim J.A., Kim Y.H. (2023). A Simple and Rapid Extraction of Lipids in Plasma Using Spin Column with Superabsorbent Polymer Beads for Mass Spectrometry. J. Anal. Sci. Technol..

[47] Silva Dos Reis A., Santos A.S., Francisco De Carvalho Gonçalves J. (2021). Ultrasound-Assisted Lipid Extractions, Enriched with Sterols and Tetranortriterpenoids, from: Carapa Guianensis Seeds and the Application of Lipidomics Using GC/MS. RSC Adv..

[48] Xie Y., Wu B., Wu Z., Tu X., Xu S., Lv X., Yin H., Xiang J., Chen H., Wei F. (2020). Ultrasound-Assisted One-Phase Solvent Extraction Coupled with Liquid Chromatography-Quadrupole Time-of-Flight Mass Spectrometry for Efficient Profiling of Egg Yolk Lipids. Food Chem..

[49] Wang Q., Oshita K., Takaoka M., Shiota K. (2021). Influence of Water Content and Cell Disruption on Lipid Extraction Using Subcritical Dimethyl Ether in Wet Microalgae. Bioresour. Technol..

[50] Zhou J., Wang M., Saraiva J.A., Martins A.P., Pinto C.A., Prieto M.A., Simal-Gandara J., Cao H., Xiao J., Barba F.J. (2022). Extraction of Lipids from Microalgae Using Classical and Innovative Approaches. Food Chem..

[51] Kanda H., Fukuta Y., Wahyudiono, Goto M. (2021). Enhancement of Lipid Extraction from Soya Bean by Addition of Dimethyl Ether as Entrainer into Supercritical Carbon Dioxide. Foods.

[52] Fernández-Acosta K., Salmeron I., Chavez-Flores D., Perez-Reyes I., Ramos V., Ngadi M., Kwofie E.M., Perez-Vega S. (2019). Evaluation of Different Variables on the Supercritical CO_2_ Extraction of Oat (*Avena sativa* L.) Oil; Main Fatty Acids, Polyphenols, and Antioxidant Content. J. Cereal Sci..

[53] Belayneh H.D., Wehling R.L., Reddy A.K., Cahoon E.B., Ciftci O.N. (2017). Ethanol-Modified Supercritical Carbon Dioxide Extraction of the Bioactive Lipid Components of Camelina Sativa Seed. J. Am. Oil Chem. Soc..

[54] Apffel A., Zhao L., Sartain M.J. (2021). A Novel Solid Phase Extraction Sample Preparation Method for Lipidomic Analysis of Human Plasma Using Liquid Chromatography/Mass Spectrometry. Metabolites.

[55] Reyes-Garcés N., Gionfriddo E. (2019). Recent Developments and Applications of Solid Phase Microextraction as a Sample Preparation Approach for Mass-Spectrometry-Based Metabolomics and Lipidomics. TrAC Trends Anal. Chem..

[56] Ramani Venkata A., Ramesh M. (2021). A Concise Review on Lipidomics Analysis in Biological Samples. ADMET DMPK.

[57] Giera M. (2015). Bioanalytical Derivatization: Is There Still Room for Development?. Bioanalysis.

[58] El-Maghrabey M.H., Kishikawa N., Kuroda N. (2020). Current Trends in Isotope-Coded Derivatization Liquid Chromatographic-Mass Spectrometric Analyses with Special Emphasis on Their Biomedical Application. Biomed. Chromatogr..

[59] Li M., Yang L., Bai Y., Liu H. (2014). Analytical Methods in Lipidomics and Their Applications. Anal. Chem..

[60] Gowda D., Li Y., Gowda S.G.B., Ohno M., Chiba H., Hui S.P. (2022). Determination of Short-Chain Fatty Acids by N,N-Dimethylethylenediamine Derivatization Combined with Liquid Chromatography/Mass Spectrometry and Their Implication in Influenza Virus Infection. Anal. Bioanal. Chem..

[61] Fu H., Zhang Q., Huang X., Ma Z., Zheng X., Li S., Duan H., Sun X., Lin F., Zhao L. (2020). A Rapid and Convenient Derivatization Method for Quantitation of Short-Chain Fatty Acids in Human Feces by Ultra-Performance Liquid Chromatography/Tandem Mass Spectrometry. Rapid Commun. Mass Spectrom..

[62] Wang F.H., Guo X.F., Fan Y.C., Tang H.B., Liang W., Wang H. (2022). Determination of Trans-Fatty Acids in Food Samples Based on the Precolumn Fluorescence Derivatization by High Performance Liquid Chromatography. J. Sep. Sci..

[63] Kvasnička A., Najdekr L., Dobešová D., Piskláková B., Ivanovová E., Friedecký D. (2023). Clinical Lipidomics in the Era of the Big Data. Clin. Chem. Lab. Med..

[64] Lia J., Vosegaard T., Guo Z. (2017). Applications of nuclear magnetic resonance in lipid analyses: An emerging powerful tool for lipidomics studies. Prog. Lipid Res..

[65] Swinnen J.V., Dehairs J. (2022). A Beginner’s Guide to Lipidomics. Biochem.

[66] Holčapek M., Liebisch G., Ekroos K. (2020). Lipidomic Analysis. Anal. Bioanal. Chem..

[67] Hyötyläinen T., Orešič M. (2016). Bioanalytical Techniques in Nontargeted Clinical Lipidomics. Bioanalysis.

[68] Höring M., Ejsing C.S., Hermansson M., Liebisch G. (2019). Quantification of Cholesterol and Cholesteryl Ester by Direct Flow Injection High-Resolution Fourier Transform Mass Spectrometry Utilizing Species-Specific Response Factors. Anal. Chem..

[69] Nielsen I.Ø., Vidas Olsen A., Dicroce-Giacobini J., Papaleo E., Andersen K.K., Jäättelä M., Maeda K., Bilgin M. (2020). Comprehensive Evaluation of a Quantitative Shotgun Lipidomics Platform for Mammalian Sample Analysis on a High-Resolution Mass Spectrometer. J. Am. Soc. Mass Spectrom..

[70] Hu C., Duan Q., Han X. (2020). Strategies to Improve/Eliminate the Limitations in Shotgun Lipidomics. Proteomics.

[71] Su B., Bettcher L.F., Hsieh W.Y., Hornburg D., Pearson M.J., Blomberg N., Giera M., Snyder M.P., Raftery D., Bensinger S.J. (2021). A DMS Shotgun Lipidomics Workflow Application to Facilitate High-Throughput, Comprehensive Lipidomics. J. Am. Soc. Mass Spectrom..

[72] Marques C., Liu L., Duncan K.D., Lanekoff I. (2022). A Direct Infusion Probe for Rapid Metabolomics of Low-Volume Samples. Anal. Chem..

[73] Xu T., Li H., Feng D., Dou P., Shi X., Hu C., Xu G. (2021). Lipid Profiling of 20 Mammalian Cells by Capillary Microsampling Combined with High-Resolution Spectral Stitching Nanoelectrospray Ionization Direct-Infusion Mass Spectrometry. Anal. Chem..

[74] Chung H.J., Lee H., Na G., Jung H., Kim D.G., Shin S.I., Jung S.E., Choi I.D., Lee J.H., Sim J.H. (2020). Metabolic and Lipidomic Profiling of Vegetable Juices Fermented with Various Probiotics. Biomolecules.

[75] Bukowski M.R., Picklo M.J. (2021). Simple, Rapid Lipidomic Analysis of Triacylglycerols in Bovine Milk by Infusion-Electrospray Mass Spectrometry. Lipids.

[76] Ledoux L., Zirem Y., Renaud F., Duponchel L., Salzet M., Ogrinc N., Fournier I. (2023). Comparing MS Imaging of Lipids by WALDI and MALDI: Two Technologies for Evaluating a Common Ground Truth in MS Imaging. Analyst.

[77] Sommella E., Salviati E., Caponigro V., Grimaldi M., Musella S., Bertamino A., Cacace L., Palladino R., Di Mauro G., Marini F. (2022). MALDI Mass Spectrometry Imaging Highlights Specific Metabolome and Lipidome Profiles in Salivary Gland Tumor Tissues. Metabolites.

[78] Li D., Ouyang Z., Ma X. (2023). Mass Spectrometry Imaging for Single-Cell or Subcellular Lipidomics: A Review of Recent Advancements and Future Development. Molecules.

[79] Khan S., Andrew R., Griffiths W., Wang Y. (2020). Mass Spectrometry Imaging of Lipids. Lipidomics: Current and Emerging Techniques.

[80] Qin S., Miao D., Zhang X., Zhang Y., Bai Y. (2023). Methods Developments of Mass Spectrometry Based Single Cell Metabolomics. TrAC Trends Anal. Chem..

[81] Hu R., Li Y., Yang Y., Liu M. (2023). Mass Spectrometry-Based Strategies for Single-Cell Metabolomics. Mass Spectrom. Rev..

[82] Jia F., Zhao X., Zhao Y. (2023). Advancements in ToF-SIMS Imaging for Life Sciences. Front. Chem..

[83] Ren J., Li H.-W., Chen L., Zhang M., Liu Y.-X., Zhang B.-W., Xu R., Miao Y.-Y., Xu X.-M., Hua X. (2023). Mass Spectrometry Imaging-Based Single-Cell Lipidomics Profiles Metabolic Signatures of Heart Failure. Research.

[84] Nabi M.M., Mamun M.A., Islam A., Hasan M.M., Waliullah A.S.M., Tamannaa Z., Sato T., Kahyo T., Setou M. (2021). Mass Spectrometry in the Lipid Study of Cancer. Expert Rev. Proteom..

[85] dos Santos A.C.A., Vuckovic D. (2024). Current Status and Advances in Untargeted LC-MS Tissue Lipidomics Studies in Cardiovascular Health. TrAC Trends Anal. Chem..

[86] Martín-Saiz L., Abad-García B., Solano-Iturri J.D., Mosteiro L., Martín-Allende J., Rueda Y., Pérez-Fernández A., Unda M., Coterón-Ochoa P., Goya A. (2023). Using the Synergy between HPLC-MS and MALDI-MS Imaging to Explore the Lipidomics of Clear Cell Renal Cell Carcinoma. Anal. Chem..

[87] Mamun A., Islam A., Eto F., Sato T., Kahyo T., Setou M. (2020). Mass Spectrometry-Based Phospholipid Imaging: Methods and Findings. Expert. Rev. Proteom..

[88] Bowman A.P., Heeren R.M.A., Ellis S.R. (2019). Advances in Mass Spectrometry Imaging Enabling Observation of Localised Lipid Biochemistry within Tissues. TrAC Trends Anal. Chem..

[89] Qi K., Wu L., Liu C., Pan Y. (2021). Recent Advances of Ambient Mass Spectrometry Imaging and Its Applications in Lipid and Metabolite Analysis. Metabolites.

[90] He M.J., Pu W., Wang X., Zhang W., Tang D., Dai Y. (2022). Comparing DESI-MSI and MALDI-MSI Mediated Spatial Metabolomics and Their Applications in Cancer Studies. Front. Oncol..

[91] Nguyen S.N., Kyle J.E., Dautel S.E., Sontag R., Luders T., Corley R., Ansong C., Carson J., Laskin J. (2019). Lipid Coverage in Nanospray Desorption Electrospray Ionization Mass Spectrometry Imaging of Mouse Lung Tissues. Anal. Chem..

[92] Khamehgir-Silz P., Gerbig S., Volk N., Schulz S., Spengler B., Hecker M., Wagner A.H. (2022). Comparative Lipid Profiling of Murine and Human Atherosclerotic Plaques Using High-Resolution MALDI MSI. Pflugers Arch..

[93] Moerman A.M., Visscher M., Slijkhuis N., Van Gaalen K., Heijs B., Klein T., Burgers P.C., De Rijke Y.B., Van Beusekom H.M.M., Luider T.M. (2021). Lipid Signature of Advanced Human Carotid Atherosclerosis Assessed by Mass Spectrometry Imaging. J. Lipid Res..

[94] Rocha B., Cillero-Pastor B., Ruiz-Romero C., Paine M.R.L., Cañete J.D., Heeren R.M.A., Blanco F.J. (2021). Identification of a Distinct Lipidomic Profile in the Osteoarthritic Synovial Membrane by Mass Spectrometry Imaging. Osteoarthr. Cartil.

[95] Denti V., Mahajneh A., Capitoli G., Clerici F., Piga I., Pagani L., Chinello C., Bolognesi M.M., Paglia G., Galimberti S. (2021). Lipidomic Typing of Colorectal Cancer Tissue Containing Tumour-Infiltrating Lymphocytes by MALDI Mass Spectrometry Imaging. Metabolites.

[96] Chen Y., Wang T., Xie P., Song Y., Wang J., Cai Z. (2021). Mass Spectrometry Imaging Revealed Alterations of Lipid Metabolites in Multicellular Tumor Spheroids in Response to Hydroxychloroquine. Anal. Chim. Acta.

[97] Visscher M., Moerman A.M., Burgers P.C., Van Beusekom H.M.M., Luider T.M., Verhagen H.J.M., Van der Steen A.F.W., Van der Heiden K., Van Soest G. (2019). Data Processing Pipeline for Lipid Profiling of Carotid Atherosclerotic Plaque with Mass Spectrometry Imaging. J. Am. Soc. Mass Spectrom..

[98] Lukowski J., Pamreddy A., Velickovic D., Zhang G., Pasa-Tolic L., Alexandrov T., Sharma K., Anderton C.R. (2020). Storage conditions of human kidney tissue sections affect spatial lipidomics analysis reproducibility. J. Am. Soc. Mass Spectrom..

[99] Dimovska Nilsson K., Karagianni A., Kaya I., Henricsson M., Fletcher J.S. (2021). (CO_2_)n+, (H_2_O)n+, and (H_2_O)n+ (CO_2_) Gas Cluster Ion Beam Secondary Ion Mass Spectrometry: Analysis of Lipid Extracts, Cells, and Alzheimer’s Model Mouse Brain Tissue. Anal. Bioanal. Chem..

[100] Fu T., Knittelfelder O., Geffard O., Clément Y., Testet E., Elie N., Touboul D., Abbaci K., Shevchenko A., Lemoine J. (2021). Shotgun Lipidomics and Mass Spectrometry Imaging Unveil Diversity and Dynamics in Gammarus Fossarum Lipid Composition. iScience.

[101] Sämfors S., Ståhlman M., Klevstig M., Borén J., Fletcher J.S. (2019). Localised Lipid Accumulation Detected in Infarcted Mouse Heart Tissue Using ToF-SIMS. Int. J. Mass Spectrom..

[102] Seubnooch P., Montani M., Tsouka S., Claude E., Rafiqi U., Perren A., Dufour J.F., Masoodi M. (2023). Characterisation of Hepatic Lipid Signature Distributed across the Liver Zonation Using Mass Spectrometry Imaging. JHEP Rep..

[103] Slijkhuis N., Towers M., Mirzaian M., Korteland S.A., Heijs B., van Gaalen K., Nieuwenhuizen I., Nigg A., van der Heiden K., de Rijke Y.B. (2023). Identifying Lipid Traces of Atherogenic Mechanisms in Human Carotid Plaque. Atherosclerosis.

[104] Sun B., Jiang S., Li M., Zhang Y., Zhou Y., Wei X., Wang H., Si N., Bian B., Zhao H. (2022). Lipidomics Combined with Transcriptomic and Mass Spectrometry Imaging Analysis of the Asiatic Toad (Bufo Gargarizans) during Metamorphosis and Bufadienolide Accumulation. Chin. Med..

[105] Henderson F., Jones E., Denbigh J., Christie L., Chapman R., Hoyes E., Claude E., Williams K.J., Roncaroli F., McMahon A. (2020). 3D DESI-MS Lipid Imaging in a Xenograft Model of Glioblastoma: A Proof of Principle. Sci. Rep..

[106] Cordeiro F.B., Jarmusch A.K., León M., Ferreira C.R., Pirro V., Eberlin L.S., Hallett J., Miglino M.A., Cooks R.G. (2020). Mammalian Ovarian Lipid Distributions by Desorption Electrospray Ionization–Mass Spectrometry (DESI-MS) Imaging. Anal. Bioanal. Chem..

[107] León M., Ferreira C.R., Eberlin L.S., Jarmusch A.K., Pirro V., Rodrigues A.C.B., Favaron P.O., Miglino M.A., Cooks R.G. (2019). Metabolites and Lipids Associated with Fetal Swine Anatomy via Desorption Electrospray Ionization—Mass Spectrometry Imaging. Sci. Rep..

[108] Sanders J.D., Shields S.W., Escobar E.E., Lanzillotti M.B., Butalewicz J.P., James V.K., Blevins M.S., Sipe S.N., Brodbelt J.S. (2022). Enhanced Ion Mobility Separation and Characterization of Isomeric Phosphatidylcholines Using Absorption Mode Fourier Transform Multiplexing and Ultraviolet Photodissociation Mass Spectrometry. Anal. Chem..

[109] Kaszycki J.L., La Rotta A., Colsch B., Fenaille F., Dauly C., Kamleh A., Wu C. (2019). Separation of Biologically Relevant Isomers on an Orbitrap Mass Spectrometer Using High-Resolution Drift Tube Ion Mobility and Varied Drift Gas Mixtures. Rapid Commun. Mass Spectrom..

[110] Lacalle-Bergeron L., Goterris-Cerisuelo R., Beltran J., Sancho J.V., Navarro-Moreno C., Martinez-Garcia F., Portolés T. (2023). Untargeted Metabolomics Approach Using UHPLC-IMS-QTOF MS for Surface Body Samples to Identify Low-Volatility Chemosignals Related to Maternal Care in Mice. Talanta.

[111] Rose B.S., Leaptrot K.L., Harris R.A., Sherrod S.D., May J.C., McLean J.A. (2021). High Confidence Shotgun Lipidomics Using Structurally Selective Ion Mobility-Mass Spectrometry. Methods Mol. Biol..

[112] Hoffmann N., Mayer G., Has C., Kopczynski D., Machot F.A., Schwudke D., Ahrends R., Marcus K., Eisenacher M., Turewicz M. (2022). A Current Encyclopedia of Bioinformatics Tools, Data Formats and Resources for Mass Spectrometry Lipidomics. Metabolites.

[113] Paglia G., Smith A.J., Astarita G. (2022). Ion Mobility Mass Spectrometry in the Omics Era: Challenges and Opportunities for Metabolomics and Lipidomics. Mass Spectrom. Rev..

[114] Zandkarimi F., Brown L.M. (2019). Application of Ion Mobility Mass Spectrometry in Lipidomics. Adv. Exp. Med. Biol..

[115] Chouinard C.D., Nagy G., Smith R.D., Baker E.S. (2019). Ion Mobility-Mass Spectrometry in Metabolomic, Lipidomic, and Proteomic Analyses.

[116] Tu J., Zhou Z., Li T., Zhu Z.J. (2019). The Emerging Role of Ion Mobility-Mass Spectrometry in Lipidomics to Facilitate Lipid Separation and Identification. TrAC Trends Anal. Chem..

[117] D’Atri V., Causon T., Hernandez-Alba O., Mutabazi A., Veuthey J.L., Cianferani S., Guillarme D. (2018). Adding a New Separation Dimension to MS and LC–MS: What Is the Utility of Ion Mobility Spectrometry?. J. Sep. Sci..

[118] Zhou Z., Shen X., Chen X., Tu J., Xiong X., Zhu Z.J. (2019). LipidIMMS Analyzer: Integrating Multi-Dimensional Information to Support Lipid Identification in Ion Mobility—Mass Spectrometry Based Lipidomics. Bioinformatics.

[119] Wu B., Wei F., Xu S., Xie Y., Lv X., Chen H., Huang F. (2021). Mass Spectrometry-Based Lipidomics as a Powerful Platform in Foodomics Research. Trends Food Sci. Technol..

[120] Zhu P., Bu G., Hu R., Ruan X., Fu R., Zhang Z., Wan Q., Liu X., Miao Y., Chen S. (2023). Lipidomic Characterization of Oocytes at Single-Cell Level Using Nanoflow Chromatography-Trapped Ion Mobility Spectrometry-Mass Spectrometry. Molecules.

[121] Lerner R., Baker D., Schwitter C., Neuhaus S., Hauptmann T., Post J.M., Kramer S., Bindila L. (2023). Four-Dimensional Trapped Ion Mobility Spectrometry Lipidomics for High Throughput Clinical Profiling of Human Blood Samples. Nat. Commun..

[122] Jiang L.X., Hernly E., Hu H., Hilger R.T., Neuweger H., Yang M., Laskin J. (2023). Nanospray Desorption Electrospray Ionization (Nano-DESI) Mass Spectrometry Imaging with High Ion Mobility Resolution. J. Am. Soc. Mass. Spectrom..

[123] Merciai F., Musella S., Sommella E., Bertamino A., D’Ursi A.M., Campiglia P. (2022). Development and Application of a Fast Ultra-High Performance Liquid Chromatography-Trapped Ion Mobility Mass Spectrometry Method for Untargeted Lipidomics. J. Chromatogr. A.

[124] Vasilopoulou C.G., Sulek K., Brunner A.D., Meitei N.S., Schweiger-Hufnagel U., Meyer S.W., Barsch A., Mann M., Meier F. (2020). Trapped Ion Mobility Spectrometry and PASEF Enable In-Depth Lipidomics from Minimal Sample Amounts. Nat. Commun..

[125] Romsdahl T.B., Cocuron J.C., Pearson M.J., Alonso A.P., Chapman K.D. (2022). A Lipidomics Platform to Analyze the Fatty Acid Compositions of Non-Polar and Polar Lipid Molecular Species from Plant Tissues: Examples from Developing Seeds and Seedlings of Pennycress (*Thlaspi arvense*). Front. Plant Sci..

[126] Lísa M., Cífková E., Khalikova M., Ovčačíková M., Holčapek M. (2017). Lipidomic Analysis of Biological Samples: Comparison of Liquid Chromatography, Supercritical Fluid Chromatography and Direct Infusion Mass Spectrometry Methods. J. Chromatogr. A.

[127] Avela H.F., Sirén H. (2020). Advances in Analytical Tools and Current Statistical Methods Used in Ultra-High-Performance Liquid Chromatography-Mass Spectrometry of Glycero-, Glycerophospho- and Sphingolipids. Int. J. Mass Spectrom..

[128] Rustam Y.H., Reid G.E. (2018). Analytical Challenges and Recent Advances in Mass Spectrometry Based Lipidomics. Anal. Chem..

[129] Avela H.F., Sirén H. (2020). Advances in Lipidomics. Clin. Chim. Acta.

[130] Züllig T., Köfeler H.C. (2021). High Resolution Mass Spectrometry in Lipidomics. Mass Spectrom. Rev..

[131] Ovčačíková M., Lísa M., Cífková E., Holčapek M. (2016). Retention Behavior of Lipids in Reversed-Phase Ultrahigh-Performance Liquid Chromatography-Electrospray Ionization Mass Spectrometry. J. Chromatogr. A.

[132] Chen L., Dean B., Liang X. (2021). A Technical Overview of Supercritical Fluid Chromatography-Mass Spectrometry (SFC-MS) and Its Recent Applications in Pharmaceutical Research and Development. Drug Discov. Today Technol..

[133] Soga T. (2023). Advances in Capillary Electrophoresis Mass Spectrometry for Metabolomics. TrAC Trends Anal. Chem..

[134] Wolrab D., Peterka O., Chocholoušková M., Holčapek M. (2022). Ultrahigh-Performance Supercritical Fluid Chromatography / Mass Spectrometry in the Lipidomic Analysis. TrAC Trends Anal. Chem..

[135] Hayasaka R., Tabata S., Hasebe M., Ikeda S., Ohnuma S., Mori M., Soga T., Tomita M., Hirayama A. (2021). Metabolomic Analysis of Small Extracellular Vesicles Derived from Pancreatic Cancer Cells Cultured under Normoxia and Hypoxia. Metabolites.

[136] Wu Z., Bagarolo G.I., Thoröe-Boveleth S., Jankowski J. (2020). “Lipidomics”: Mass Spectrometric and Chemometric Analyses of Lipids. Adv. Drug Deliv. Rev..

[137] Wu Z., Shon J.C., Liu K.-H. (2014). Mass Spectrometry-Based Lipidomics and Its Application ToBiomedical Research. J. Lifestyle Med..

[138] Zeng K., Zhou X., Liu W., Nie C., Zhang Y. (2023). Determination of Endogenous Sphingolipid Content in Stroke Rats and HT22 Cells Subjected to Oxygen-Glucose Deprivation by LC—MS/MS. Lipids Health Dis..

[139] Chang J.K., Teo G., Pewzner-Jung Y., Cuthbertson D.J., Futerman A.H., Wenk M.R., Choi H., Torta F. (2023). Q-RAI Data-Independent Acquisition for Lipidomic Quantitative Profiling. Sci. Rep..

[140] Zhou H., Nong Y., Zhu Y., Liang Y., Zhang J., Chen H., Zhu P., Zhang Q. (2022). Serum Untargeted Lipidomics by UHPLC-ESI-HRMS Aids the Biomarker Discovery of Colorectal Adenoma. BMC Cancer.

[141] Mocciaro G., D’amore S., Jenkins B., Kay R., Murgia A., Herrera-marcos L.V., Neun S., Sowton A.P., Hall Z., Palma-Duran S.A. (2022). Lipidomic Approaches to Study HDL Metabolism in Patients with Central Obesity Diagnosed with Metabolic Syndrome. Int. J. Mol. Sci..

[142] Xu Y., Li H., Han Y., Wang T., Wang Y., Gong J., Gao K., Chen W., Li W., Zhang H. (2022). A Simple and Rapid Method for Extraction and Measurement of Circulating Sphingolipids Using LC–MS/MS: A Targeted Lipidomic Analysis. Anal. Bioanal. Chem..

[143] Duan L., Scheidemantle G., Lodge M., Cummings M.J., Pham E., Wang X., Kennedy A., Liu X. (2022). Prioritize Biologically Relevant Ions for Data-Independent Acquisition (BRI-DIA) in LC–MS/MS-Based Lipidomics Analysis. Metabolomics.

[144] Gao X., Hu X.H., Zhang Q., Wang X.J., Wen X.H., Wang Y., Zhang Y.X., Sun W.J. (2021). Exploring Lipid Biomarkers of Coronary Heart Disease for Elucidating the Biological Effects of Gelanxinning Capsule by Lipidomics Method Based on LC–MS. Biomed. Chromatogr..

[145] Lísa M., Řehulková H., Hančová E., Řehulka P. (2021). Lipidomic Analysis Using Hydrophilic Interaction Liquid Chromatography Microgradient Fractionation of Total Lipid Extracts. J. Chromatogr. A.

[146] Yazd H.S., Bazargani S.F., Vanbeek C.A., King-Morris K., Heldermon C., Segal M.S., Clapp W.L., Garrett T.J. (2021). LC-MS Lipidomics of Renal Biopsies for the Diagnosis of Fabry Disease. J. Mass Spectrom. Adv. Clin. Lab..

[147] Nakashima Y., Sakai Y., Mizuno Y., Furuno K., Hirono K., Takatsuki S., Suzuki H., Onouchi Y., Kobayashi T., Tanabe K. (2021). Lipidomics Links Oxidized Phosphatidylcholines and Coronary Arteritis in Kawasaki Disease. Cardiovasc. Res..

[148] Nishida-Aoki N., Izumi Y., Takeda H., Takahashi M., Ochiya T., Bamba T. (2020). Lipidomic Analysis of Cells and Extracellular Vesicles from High-and Low-Metastatic Triple-Negative Breast Cancer. Metabolites.

[149] López-Bascón M.A., Calderón-Santiago M., Díaz-Lozano A., Camargo A., López-Miranda J., Priego-Capote F. (2020). Development of a Qualitative/Quantitative Strategy for Comprehensive Determination of Polar Lipids by LC–MS/MS in Human Plasma. Anal. Bioanal. Chem..

[150] Leung H.H., Leung K.S., Durand T., Galano J.M., Lee J.C.Y. (2020). Measurement of Enzymatic and Nonenzymatic Polyunsaturated Fatty Acid Oxidation Products in Plasma and Urine of Macular Degeneration Using LC-QTOF-MS/MS. Lipids.

[151] Williams J., Zhu K., Crampon E., Iffland A. (2020). Fit-for-Purpose Biomarker LC-MS/MS Qualification for the Quantitation of Very Long Chain Fatty Acids in Human Cerebrospinal Fluid. Bioanalysis.

[152] Gong L.L., Yang S., Zhang W., Han F.F., Lv Y.L., Xuan L.L., Liu H., Liu L. (2020). hong Discovery of Metabolite Profiles of Metabolic Syndrome Using Untargeted and Targeted LC–MS Based Lipidomics Approach. J. Pharm. Biomed. Anal..

[153] Liu X., Zhang M., Cheng X., Liu X., Sun H., Guo Z., Li J., Tang X., Wang Z., Sun W. (2020). LC-MS-Based Plasma Metabolomics and Lipidomics Analyses for Differential Diagnosis of Bladder Cancer and Renal Cell Carcinoma. Front. Oncol..

[154] Takashima S., Toyoshi K., Yamamoto T., Shimozawa N. (2020). Positional Determination of the Carbon–Carbon Double Bonds in Unsaturated Fatty Acids Mediated by Solvent Plasmatization Using LC–MS. Sci. Rep..

[155] Pousinis P., Gowler P.R.W., Burston J.J., Ortori C.A., Chapman V., Barrett D.A. (2020). Lipidomic Identification of Plasma Lipids Associated with Pain Behaviour and Pathology in a Mouse Model of Osteoarthritis. Metabolomics.

[156] Zhang Q., Xu H., Liu R., Gao P., Yang X., Jin W., Zhang Y., Bi K., Li Q. (2019). A Novel Strategy for Targeted Lipidomics Based on LC-Tandem-MS Parameters Prediction, Quantification, and Multiple Statistical Data Mining: Evaluation of Lysophosphatidylcholines as Potential Cancer Biomarkers. Anal. Chem..

[157] Liu T., Peng F., Yu J., Tan Z., Rao T., Chen Y., Wang Y., Liu Z., Zhou H., Peng J. (2019). LC-MS-Based Lipid Profile in Colorectal Cancer Patients: TAGs Are the Main Disturbed Lipid Markers of Colorectal Cancer Progression. Anal. Bioanal. Chem..

[158] Vu N., Narvaez-Rivas M., Chen G.Y., Rewers M.J., Zhang Q. (2019). Accurate Mass and Retention Time Library of Serum Lipids for Type 1 Diabetes Research. Anal. Bioanal. Chem..

[159] Meierhofer D. (2019). Acylcarnitine Profiling by Low-Resolution LC-MS. PLoS ONE.

[160] Ma S.R., Tong Q., Zhao Z.X., Cong L., Yu J.B., Fu J., Han P., Pan L.B., Gu R., Peng R. (2019). Determination of Berberine-Upregulated Endogenous Short-Chain Fatty Acids through Derivatization by 2-Bromoacetophenone. Anal. Bioanal. Chem..

[161] Dasilva G., Muñoz S., Lois S., Medina I. (2019). Non-Targeted LC-MS/MS Assay for Screening over 100 Lipid Mediators from ARA, EPA, and DHA in Biological Samples Based on Mass Spectral Fragmentations. Molecules.

[162] Maekawa M., Jinnoh I., Matsumoto Y., Narita A., Mashima R., Takahashi H., Iwahori A., Saigusa D., Fujii K., Abe A. (2019). Structural Determination of Lysosphingomyelin-509 and Discovery of Novel Class Lipids from Patients with Niemann–Pick Disease Type C. Int. J. Mol. Sci..

[163] King A., Baginski M., Morikawa Y., Rainville P.D., Gethings L.A., Wilson I.D., Plumb R.S. (2019). Application of a Novel Mass Spectral Data Acquisition Approach to Lipidomic Analysis of Liver Extracts from Sitaxentan-Treated Liver-Humanized PXB Mice. J. Proteome Res..

[164] Körber T.T., Sitz T., Abdalla M.A., Mühling K.H., Rohn S. (2023). LC-ESI-MS/MS Analysis of Sulfolipids and Galactolipids in Green and Red Lettuce (*Lactuca sativa* L.) as Influenced by Sulfur Nutrition. Int. J. Mol. Sci..

[165] Fabio Turco J., Benhur Mokochinski J., Reyes Torres Y. (2023). Lipidomic Analysis of Geopropolis of Brazilian Stingless Bees by LC-HRMS. Food Res. Int..

[166] Martin J.J.J., Wu Q., Feng M., Li R., Zhou L., Zhang S., Yang C., Cao H. (2023). Lipidomic Profiles of Lipid Biosynthesis in Oil Palm during Fruit Development. Metabolites.

[167] Wiedmaier-Czerny N., Vetter W. (2023). LC-Orbitrap-HRMS Method for Analysis of Traces of Triacylglycerols Featuring Furan Fatty Acids. Anal. Bioanal. Chem..

[168] Alves E., Rey F., Melo T., Barros M.P., Domingues P., Domingues R. (2022). Bioprospecting Bioactive Polar Lipids from Olive (*Olea europaea* cv. *Galega vulgar*) Fruit Seeds: LC-HR-MS/MS Fingerprinting and Sub-Geographic Comparison. Foods.

[169] Aurum F.S., Imaizumi T., Thammawong M., Suhandy D., Praseptiangga D., Tsuta M., Nagata M., Nakano K. (2022). Lipidomic Profiling of Indonesian Coffee to Determine Its Geographical Origin by LC–MS/MS. Eur. Food Res. Technol..

[170] Li M., Zhu M., Chai W., Wang Y., Fan D., Lv M., Jiang X., Liu Y., Wei Q., Wang C. (2021). Determination of Lipid Profiles of Dezhou Donkey Meat Using an LC-MS-Based Lipidomics Method. J. Food Sci..

[171] Kokotou M.G., Mantzourani C., Bourboula A., Mountanea O.G., Kokotos G. (2020). A Liquid Chromatography-High Resolution Mass Spectrometry (LC-HRMS) Method for the Determination of Free Hydroxy Fatty Acids in Cow and Goat Milk. Molecules.

[172] Lukić I., Ros A.D., Guella G., Camin F., Masuero D., Mulinacci N., Vrhovsek U., Mattivi F. (2020). Lipid Profiling and Stable Isotopic Data Analysis for Differentiation of Extra Virgin Olive Oils Based on Their Origin. Molecules.

[173] Claassen C., Kuballa J., Rohn S. (2019). Polar Lipids in Starch-Rich Commodities to Be Analyzed with LC-MS-Based Metabolomics—Optimization of Ionization Parameters and High-Throughput Extraction Protocols. Metabolites.

[174] Zartmann A., Völcker L., Hammann S. (2023). Quantitative Analysis of Fatty Acids and Vitamin E and Total Lipid Profiling of Dietary Supplements from the German Market. Eur. Food Res. Technol..

[175] Deschamps E., Schaumann A., Schmitz-Afonso I., Afonso C., Dé E., Loutelier-Bourhis C., Alexandre S. (2021). Membrane Phospholipid Composition of Pseudomonas Aeruginosa Grown in a Cystic Fibrosis Mucus-Mimicking Medium. Biochim. Biophys. Acta Biomembr..

[176] Bill M.K., Brinkmann S., Oberpaul M., Patras M.A., Leis B., Marner M., Maitre M.P., Hammann P.E., Vilcinskas A., Schuler S.M.M. (2021). Novel Glycerophospholipid, Lipo-and N-Acyl Amino Acids from Bacteroidetes: Isolation, Structure Elucidation and Bioactivity. Molecules.

[177] Wozny K., Lehmann W.D., Wozny M., Akbulut B.S., Brügger B. (2019). A Method for the Quantitative Determination of Glycerophospholipid Regioisomers by UPLC-ESI-MS/MS. Anal. Bioanal. Chem..

[178] Alekseyeva K.S., Mähnert B., Berthiller F., Breyer E., Herndl G.J., Baltar F. (2021). Adapting an Ergosterol Extraction Method with Marine Yeasts for the Quantification of Oceanic Fungal Biomass. J. Fungi.

[179] Flor S., Sosa Alderete L., Dobrecky C., Tripodi V., Agostini E., Lucangioli S. (2021). LC-ESI-MS/MS Method for the Profiling of Glycerophospholipids and Its Application to the Analysis of Tobacco Hairy Roots as Early Indicators of Phenol Pollution. Chromatographia.

[180] Jankevics A., Jenkins A., Dunn W.B., Najdekr L. (2021). An Improved Strategy for Analysis of Lipid Molecules Utilising a Reversed Phase C30 UHPLC Column and Scheduled MS/MS Acquisition. Talanta.

[181] McDonald J.G., Ejsing C.S., Kopczynski D., Holčapek M., Aoki J., Arita M., Arita M., Baker E.S., Ber-trand-Michel J., Bowden J.A. (2022). Introducing the Lipidomics Minimal Reporting Checklist. Nat. Metab..

[182] Guo J., Huan T. (2020). Comparison of Full-Scan, Data-Dependent, and Data-Independent Acquisition Modes in Liquid Chromatography-Mass Spectrometry Based Untargeted Metabolomics. Anal. Chem..

[183] Defossez E., Bourquin J., von Reuss S., Rasmann S., Glauser G. (2023). Eight Key Rules for Successful Data-Dependent Acquisition in Mass Spectrometry-Based Metabolomics. Mass Spectrom. Rev..

[184] Davies V., Wandy J., Weidt S., Van Der Hooft J.J.J., Miller A., Daly R., Rogers S. (2021). Rapid Development of Improved Data-Dependent Acquisition Strategies. Anal. Chem..

[185] Kirkwood K.I., Pratt B.S., Shulman N., Tamura K., MacCoss M.J., MacLean B.X., Baker E.S. (2022). Utilizing Skyline to Analyze Lipidomics Data Containing Liquid Chromatography, Ion Mobility Spectrometry and Mass Spectrometry Dimensions. Nat. Protoc..

[186] Zhang H., Liu Y., Fields L., Shi X., Huang P., Lu H., Schneider A.J., Tang X., Puglielli L., Welham N.V. (2023). Single-Cell Lipidomics Enabled by Dual-Polarity Ionization and Ion Mobility-Mass Spectrometry Imaging. Nat. Commun..

[187] Calderón C., Rubarth L., Cebo M., Merfort I., Lämmerhofer M. (2020). Lipid Atlas of Keratinocytes and Betulin Effects on Its Lipidome Profiled by Comprehensive UHPLC–MS/MS with Data Independent Acquisition Using Targeted Data Processing. Proteomics.

[188] Rombouts C., De Spiegeleer M., Van Meulebroek L., De Vos W.H., Vanhaecke L. (2019). Validated Comprehensive Metabolomics and Lipidomics Analysis of Colon Tissue and Cell Lines. Anal. Chim. Acta.

